# National Economic Development and Disparities in Body Mass Index: A Cross-Sectional Study of Data from 38 Countries

**DOI:** 10.1371/journal.pone.0099327

**Published:** 2014-06-11

**Authors:** Melissa Neuman, Ichiro Kawachi, Steven Gortmaker, SV. Subramanian

**Affiliations:** Department of Social and Behavioral Sciences, Harvard School of Public Health, Boston, Massachusetts, United States of America; Old Dominion University, United States of America

## Abstract

**Background:**

Increases in body mass index (BMI) and the prevalence of overweight in low- and middle income countries (LMICs) are often ascribed to changes in global trade patterns or increases in national income. These changes are likely to affect populations within LMICs differently based on their place of residence or socioeconomic status (SES).

**Objective:**

Using nationally representative survey data from 38 countries and national economic indicators from the World Bank and other international organizations, we estimated ecological and multilevel models to assess the association between national levels of gross domestic product (GDP), foreign direct investment (FDI), and mean tariffs and BMI.

**Design:**

We used linear regression to estimate the ecological association between average annual change in economic indicators and BMI, and multilevel linear or ordered multinomial models to estimate associations between national economic indicators and individual BMI or over- and underweight. We also included cross-level interaction terms to highlight differences in the association of BMI with national economic indicators by type of residence or socioeconomic status (SES).

**Results:**

There was a positive but non-significant association of GDP and mean BMI. This positive association of GDP and BMI was greater among rural residents and the poor. There were no significant ecological associations between measures of trade openness and mean BMI, but FDI was positively associated with BMI among the poorest respondents and in rural areas and tariff levels were negatively associated with BMI among poor and rural respondents.

**Conclusion:**

Measures of national income and trade openness have different associations with the BMI across populations within developing countries. These divergent findings underscore the complexity of the effects of development on health and the importance of considering how the health effects of “globalizing” economic and cultural trends are modified by individual-level wealth and residence.

## Introduction

In the past two decades, mean BMI has increased in lower income countries, prompting some researchers to argue that obesity and chronic disease prevention should become higher public health priorities in these areas [1]–[8]. Many authors have linked increased body weight in lower-income countries with economic development, suggesting that that “globalization,” or worldwide integration of culture, trade and foreign investment, has changed the supply and demand of food by altering trade and employment patterns and cultural norms around ideal body size [9]–[15].

Examinations of the effects of globalization on health have measured globalization using two types of national economic indicators. Some studies have assessed the associations between per capita income, measured as per capita GDP (gross domestic product) or GNP (gross national product) and BMI, with the underlying assumptions that exposure to global markets increases with national income, and that increased per capita income leads to higher BMI either through greater resources available to purchase foods or through changes in labor requirements leading to increased sedentary employment [12], [16], [17]. Ecological analyses have identified a positive association between per capita income or GNP and mean BMI and rates of overweight and obesity [18]–[20]. In contrast, other authors measure globalization using indicators of openness to trade in food and food preparation technology, often measured as rates of foreign direct investment (FDI) or annual tariffs. Openness to trade is expected to increase body mass and prevalence of obesity by increasing the availability and decreasing the price of process foods and food preparation technologies, and by exposing populations to marketing campaigns promoting processed foods [10], [13], [21], [22]. While case studies of obesity and foreign investment in processed foods in developing countries, notably China, argue that these two phenomena are linked [23], [24], recent studies found that associations between levels of overall FDI, mean tariffs, or GDP and prevalence of overweight were positive but not significant among most populations across a selection of low- and middle-income countries [25], [26].

The current discourse on globalization and obesity focuses on national-level trends in dietary consumption and body weight; however, broad changes in food availability and consumption will have differential effects on individuals within populations based on their geographic location and socioeconomic status [27]. Social and geographic literature on globalization emphasizes that economic polarization and spatial segregation, both between and within countries, accompanies the internationalization of trade [28], [29]. Authors who have considered the public health effects of globalization have also emphasized its potential for increasing economic inequality and exacerbating health disparities [30], [31].

Urban-rural differentials are particularly important to consider in the context of globalization because of the role cities play as nodes for international trade and culture [32], [33]. Evidence on urban-rural differentials in mean BMI and obesity in developing countries generally suggests that urban residents are heavier than rural residents [17], [34]–[36], but that, in higher income countries, mean body weight of urban and rural populations is similar [37]. Several authors have investigated the association of national-level urbanization with BMI. One study using data from 70 countries across the development spectrum found inverse associations between SES and BMI within more urbanized countries [38]. However, this study did not adjust for national economic development, which is likely to be correlated with urbanization and is also likely to affect body weight. A study adjusting for national GDP found no association between national-level urbanization percent and prevalence of overweight [39]. More importantly, studies that only consider urbanization as a national-level construct miss the opportunity to illuminate and quantify urban-rural disparities in health, and to more fully capture the effects of broad economic changes on individual health.

Because economic development associated with globalization is also likely to differentially affect wealthy and poor populations, it is also important to consider changes in socioeconomic differentials in health as a possible outcome of globalization. While studies in a few middle-income countries have identified null or inverse associations between SES and BMI or overweight, particularly within urban areas of developing countries [18], [35], recent cross-national analyses have shown that, in general, there is a persistent and positive association between socioeconomic status (SES) and BMI in developing countries [40]–[42]. Moreover, many assessments of the direction and strength of the association between SES and BMI or overweight are based on urban samples only [18], [43]–[45]. Given that global changes are likely to have differential effects in rural areas and among the poor, it is especially important to assess the differential impact of “globalization” measures across locations and socioeconomic group.

## Data and Methods

This analysis uses ecological and multilevel analyses to assess associations between changes in national-level GDP, foreign direct investment (FDI), and tariffs on BMI and prevalence of over and underweight among adult women. Individual-level data for this study came from Demographic and Health Surveys (DHS) of women of reproductive age (15–49 yrs.) conducted in 38 countries at two time periods between 1991 and 2010 [46]. To select surveys for inclusion in the analysis, we identified the earliest and latest survey from countries where two or more surveys had been fielded after 1990. The DHS are household sample surveys measuring indicators of population health, maternal and child health, and nutrition [47]. The target population in these DHS surveys included all women or ever-married women of reproductive age, with either the full sample or a subsample of women selected for anthropometric measurements.

DHS surveys employ extensive interviewer training, standardized measurement tools and techniques, an identical core questionnaire, and instrument pretesting to ensure standardization and comparability across diverse sites and time (see www.measuredhs.com/pubs/pdf/DHSG4/Recode4DHS.pdf) [48]. The surveys use a multistage stratified design with probabilistic sampling, with each elementary unit having a defined probability of selection [49]. Each survey was stratified by urban and rural status and by country-specific geographic or administrative regions. Detailed sampling plans are available from survey final reports at www.measuredhs.com/pubs/search/search_results.cfm?Type=5&srchTp=type&newSrch=1 [50]. Response rates for the surveys are generally high, ranging between 88–99% of households and 85–95% of women within households [51]. Because the surveys collect representative data, and have been using a similar survey protocol for the past 20 years, the DHS is a valuable data source for studying population health across developing countries [52]–[54].

National-level data on gross domestic product, foreign direct investment, and net national commodity inflows were taken from a variety of academic and international databases. **Table 1** lists the sources of each of these data.

**Table 1:** Sources for macroeconomic data.

Data	Description	Source
Gross DomesticProduct (GDP)	PPP Converted GDP per capita (Laspeyres) at 2005 constantprices, averaged data for year of survey and five prior years.	Penn World Tables [82]
Foreign Direct Investment(FDI)	Inward foreign direct investment flows, annual, in 000 000s,averaged data for year of survey and five prior years. (Enteredinto models as percent GDP normalized to 2005 dollars)	United Nations Commission on Trade and Development (UNCTAD) [83], [84]
Average tariffrate	Import duties as a percent of total import values for year ofsurvey.	World Integrated Trade Solution, World Bank [85]

### Study Population and Sample Size

This study uses a pooled cross-sectional design, incorporating data from 38 countries and two surveys per country. The initial sample included 1,028,441 women interviewed in 38 countries. Many surveys included anthropometry measurements on a subsample of women: 256,500 women were not included in the subsample for height and weight measurements and were excluded from the analysis. An additional 11,742 women were eligible for measurement but had missing or implausible values; these women were also excluded from analysis. Women who were pregnant at the time of the survey (n = 59,141) and women who were outside the 15–49 year age range (n = 3,777) were also excluded from analysis. An additional 120 women were missing data on other covariates used in the analysis (DHS wealth index, educational attainment, marital status) and were removed from the analysis, leaving a final analytic sample of 697,573 women (232,150 at time 1; 465,423 at time 2).

### Outcome Measures

The primary outcome for this analysis was body mass index (BMI) among non-pregnant DHS respondents ages 15–49 years. Respondent BMI was calculated as weight in kilograms divided by height in meters squared (kg/m^2^). Weight was measured by trained investigators using a solar-powered scale with accuracy of ±100g, and height was measured using an adjustable board calibrated in millimeters [48].

BMI was chosen as the primary outcome because it provides a readily available measurement of adiposity, it is comparable across countries and settings, and it has been found to be strongly correlated with the densitometry measurements of adiposity commonly cited as the “gold standard” of adiposity measurement [55]. BMI is a particularly useful outcome to consider because it encompasses the full spectrum of body weight, from under- to over-nutrition, that may be present in developing societies. Additionally, some evidence suggests that risks of coronary heart disease and all-cause mortality increase at BMI levels less than 25 kg/m^2^, particularly among persons of Asian descent [56]–[59], making standard over- and underweight cut-off points less useful for understanding disease risk in these populations. Percent overweight (BMI>25 kg/m^2^) and percent underweight (BMI<18.5 kg/m^2^) were included as secondary outcomes to encourage comparison of results with other studies using these outcomes. Overweight is a commonly recognized marker of individual chronic disease risk [60], and underweight is a well-recognized indicator of poor nutrition in developing countries.

For individual-level analyses, the outcome variables used were either measured BMI or over- and underweight status of individuals. For ecological analyses, we calculated mean BMI or percent overweight for each country and survey year, and estimated the average annual change in national mean BMI or proportion over- or underweight. These annualized changes served as outcomes in the ecological analyses.

### Independent Variables

The primary predictors in these analyses are national per capita GDP, FDI in 2005 dollars as a percent of national GDP, and average tariff rates, entered into models as continuous variables centered around the grand mean. Because economic variables are expected to have a lagged effect on individual body weight and data on GDP and FDI were readily available, both these variables were entered into models as the average of values from the survey year and five prior years. Because average tariff data was sparse for years prior to 2000, the estimated average tariff for the year of survey only was included in models. For country and survey years where data were not reported by the World Bank, values were imputed linearly from existing data before and after the survey year within each country. Additional details on these data and data sources are provided in **table 1**. Average annual changes in each of these variables were included as predictors in ecological analyses.

Individual-level analyses included both main associations of national economic variables and interactions between national-level variables and markers of SES and urban residence. SES, as measured using a wealth index, and urban residence were included in the analysis as modifiers. Urban residents were respondents living in an urban area as defined by the national census or statistical bureau in each country at the time the survey was conducted, and is measured at the level of primary sampling unit. The primary measure of SES was an index of overall household assets. This index compares the wealth of respondents within countries by comparing the assets available within households in each country. The index was calculated using principal components analysis (PCA): z-scores for each variable measuring a household’s assets and utilities were developed, PCA was conducted on these standardized variable to identify the principal component underlying asset ownership, and the values of the indicator variables were multiplied by the factor loadings for each household and summed to produce a standardized household index value with a mean of 0 and a standard deviation of 1. This standardized score was then divided into quintiles for each country [61]–[63].

All individual-level models were also adjusted for respondent age, educational attainment, marital status, and year of survey. Respondent age was entered to the models in 5-year age categories. Educational attainment was specified as having no education or incomplete primary education, having completed primary education, or having some secondary or higher schooling. Marital status was entered as a binary variable, with ever-married also including widowed, divorced, and co-habiting women. Year of survey was entered as a categorical variable, with 1991 as reference.

### Analysis

The analysis plan included both ecological analyses assessing the association between average annual changes in national-level economic variables and changes in BMI and proportion overweight and underweight, as well as multilevel models incorporating both individual- and national-level predictors of BMI. For country-level ecological analyses, we estimated ordinary least squares regression analyses with robust standard errors, using the following single level model:
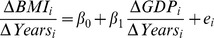



In the model above, *i* represents country. A series of ecological models were fitted. First, we estimated the association between change in GDP on changes in BMI. Next, we estimated the association between change in FDI and BMI after adjusting for GDP, and the association between change in tariffs and BMI after adjusting for GDP.

Multilevel linear analysis was used to estimate the associations of individual- and national-level predictors on BMI. We modeled these data using a four-level data structure, with individuals nested within primary sampling units (PSUs), sampling regions, and countries [64]. While the DHS samples all eligible women in sampled households, most households had only one respondent in most countries (average household size across all countries in time 1: 1.19 women; in time 2: 1.35 women.) For this reason, clustering by household was not incorporated into the data structure. Associations were estimated using the following random-intercepts model:
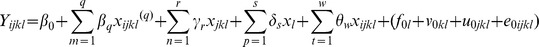



In this equation, *i,j, k,* and *l* represent the individual, sampling unit, region, and country, respectively. *Y* is the respondent’s BMI, *x^(q)^* is the *q-*th individual-level predictor, *x^(r)^* is the *r*-th PSU-level predictor, and *x^(s)^* is the *s*-th country-level predictor and *x^(w)^* is the *w*-th year indicator; *β, γ_r_,* and *δ_s_* represent the associations of individual-, PSU-, and national-level predictors, respectively; and *θ_w_* represents the association of being surveyed in year *w*. Residuals at all four levels (*f_01_*, *v_0kl_*, *u_0jkl_*, *e_0ijkl_*) are assumed to be normally distributed with mean zero. In the secondary analysis, probability of under- and overweight were modeled as outcomes using similar equations with a multinomial outcome and normal weight as the reference group.

The analysis strategy for the multilevel analyses was as follows: we first fit models including individual-level predictors such as age, educational status, and wealth index; year of survey; urban residence; and the main effect of per capita GDP. We then assessed whether the association between GDP and BMI differed by type of residence and household wealth by adding cross-level interaction terms. Finally, we assessed the main associations interacted with urban residence and wealth of FDI or average tariff rates after adjustment for individual-level covariates and per capita GDP. The secondary analysis with under- and overweight as outcomes followed the same pattern. Because a large proportion of the final sample came from two surveys conducted in India, we conducted a sensitivity analysis with data from India excluded to ensure that results were not driven primarily by these surveys. Multilevel models with the continuous BMI outcome were estimated using an interactive generalized least squares estimation algorithm. Models with the non-linear outcome used the MQL estimation procedure and first-order linearization. Descriptive statistics and ecological models were calculated using Stata SE 12.0 [65] and multilevel models were estimated using MLwiN 2.25 [66].

## Results

National-level GDP per capita, FDI, and average tariffs are summarized in **table 2**. Of the 38 countries included in this analysis, 11 had GDP per capita less than US$ 1,000 and 18 had GDP between US$ 1,000 and US$ 4,000 in the later round of surveys. The country with the highest GDP per capita was Turkey (US$ 8,465 in 2003.) In the later round of surveys, the lowest percent FDI was in Nepal (0.02% in 2006) and the highest was in Jordan (6.76% in 2007). Fifteen of 38 countries had FDI that was less than 1% of GDP, and 21 of 38 had FDI between 1% and 5% of GDP. Average tariff rates ranged from 2.80% (Haiti 1994) to 76.13% (Zimbabwe 1994) in the first time period and from 2.80% (Haiti 2005) to 30.08% (Morocco 2003) in the second time period. Average tariff rates decreased in 33 of 38 countries between time periods.

**Table 2:** National-level economic indicators by country and year.

Country	Time 1				Time 2				Average annual change		
	Year	GDP (in 000s)	FDI (% GDP)	Average tariff	Year	GDP (in000s)	FDI (%GDP)	Averagetariff	GDP	FDI	Average tariff
Armenia	2000	2.57	1.55	2.98	2005	1.46	4.54	2.86	0.39	−0.02	−0.02
Bangladesh	1996	0.86	0.07	58.40	2007	0.33	1.29	14.58	0.04	0.02	−3.98
Benin	1996	1.09	1.02	12.19	2006	0.52	1.23	11.99	0.01	−0.05	−0.02
Bolivia	1994	2.98	0.80	9.99	2008	0.55	3.74	8.32	0.05	−0.02	−0.12
Burkina Faso	1993	0.67	0.03	25.33	2003	0.16	0.86	11.98	0.02	0.01	−1.34
Cambodia	2000	1.07	2.28	16.38	2005	1.10	1.56	14.26	0.10	−0.24	−0.42
Cameroon	1998	1.63	0.35	18.15	2004	0.95	1.80	18.05	0.03	0.10	−0.02
Chad	1996	0.72	0.50	33.41	2004	6.54	1.24	18.05	0.06	0.75	−1.92
Colombia	1995	5.27	1.52	13.67	2010	2.56	7.53	12.50	0.15	0.07	−0.08
Cote d'Ivoire	1994	1.34	0.37	20.49	1998	1.18	1.34	15.79	0.00	0.20	−1.18
Egypt	1995	3.41	1.10	34.64	2008	1.83	4.82	16.94	0.11	0.06	−1.36
Ethiopia	2000	0.46	0.58	20.51	2005	1.09	0.51	17.32	0.01	0.10	−0.64
Ghana	1993	0.82	0.29	14.90	2008	1.93	1.21	13.00	0.03	0.11	−0.13
Guatemala	1995	5.05	0.28	10.05	1998	0.43	5.22	8.19	0.06	0.05	−0.62
Guinea	1999	0.75	0.40	12.18	2005	0.75	0.87	11.94	0.02	0.06	−0.04
Haiti	1994	1.41	0.07	2.80	2005	0.10	1.36	2.80	0.00	0.00	0.00
India	1998	1.69	0.17	31.52	2005	0.26	2.56	18.30	0.12	0.01	−1.89
Jordan	1997	3.81	0.50	43.24	2007	6.76	4.41	11.20	0.06	0.63	−3.20
Kazakhstan	1995	4.76	1.51	10.28	1999	1.85	4.74	6.97	0.00	0.09	−0.83
Kenya	1998	1.13	0.08	23.33	2008	0.37	1.20	12.52	0.01	0.03	−1.08
Lesotho	2004	1.25	1.76	7.97	2009	2.64	1.31	7.48	0.01	0.18	−0.10
Madagascar	1997	0.79	0.15	6.16	2008	2.63	0.81	12.45	0.00	0.23	0.57
Malawi	1992	0.56	−0.05	34.83	2010	0.76	0.65	12.84	0.01	0.05	−1.22
Mali	1995	0.71	0.39	16.34	2006	1.60	0.92	11.99	0.02	0.11	−0.40
Morocco	1992	2.38	1.22	75.80	2003	1.87	2.89	30.08	0.05	0.06	−4.16
Mozambique	1997	0.40	1.01	15.70	2003	3.62	0.56	20.16	0.03	0.43	0.74
Namibia	1992	3.91	1.00	8.09	2006	3.25	4.78	7.69	0.06	0.16	−0.03
Nepal	1996	0.99	0.02	16.37	2006	0.02	1.07	12.39	0.01	0.00	−0.40
Nicaragua	1998	1.92	1.49	5.52	2001	2.43	2.07	4.74	0.05	0.31	−0.26
Niger	1998	0.52	−0.10	12.11	2006	0.35	0.53	11.99	0.00	0.06	−0.02
Nigeria	2003	1.78	1.01	23.97	2008	1.87	1.96	12.00	0.04	0.17	−2.39
Peru	1991	4.04	0.01	19.28	2004	1.22	5.53	10.73	0.11	0.09	−0.66
Rwanda	2000	0.66	0.10	13.24	2005	0.15	0.84	18.74	0.04	0.01	1.10
Tanzania	1996	0.68	0.42	23.26	2004	1.60	0.82	13.16	0.02	0.15	−1.26
Turkey	1993	7.56	1.03	9.34	2003	0.30	8.47	9.98	0.09	−0.07	0.06
Uganda	1995	0.69	0.44	15.86	2006	1.22	1.03	12.76	0.03	0.07	−0.28
Zambia	1996	0.89	1.66	17.30	2007	3.45	1.79	13.73	0.08	0.16	−0.32
Zimbabwe	1994	0.23	0.97	76.13	2005	1.35	0.17	15.12	−0.01	0.03	−5.55

Individual BMI for each survey is summarized in **table 3**. In the later time period, mean BMI was highest in Egypt (28.85 kg/m^2^ in 2008) and Jordan (28.34 kg/m^2^ in 2007) and was lowest in Ethiopia (20.36 kg/m^2^ in 2005). Percent underweight was highest in India (29.6% in 2005) and percent overweight was highest in Egypt (76.0% in 2008). Mean BMI decreased between time periods in 6 of 38 countries, but percent overweight and percent underweight increased in 17 of 38 countries.

**Table 3:** Percent overweight and underweight by country and survey year.

Country	Time 1					Time 2					Average annual change		
	Year	N	MeanBMI(SD)	Percentunderweight	Percent overweight	Year	N	Mean BMI (SD)	Percent underweight	Percent overweight	BMI	Percentunderweight	Percent overweight
Armenia	2000	5,981	24.94	3.4%	41.5%	2005	6,067	25.19	4.7%	43.4%	0.05	0.01	0.00
			(4.76)					(5.33)					
Bangladesh	1996	4,045	18.86	51.1%	2.9%	2007	10,106	20.87	28.6%	13.7%	0.183	−0.23	−0.02
			(2.82)					(3.71)					
Benin	1996	2,330	21.13	14.8%	8.5%	2006	14,883	22.48	9.4%	17.9%	0.135	−0.05	−0.01
			(3.12)					(4.22)					
Bolivia	1994	2,346	24.22	2.3%	32.9%	2008	15,539	25.81	1.9%	49.4%	0.113	0.00	0.00
			(3.64)					(4.78)					
Burkina Faso	1993	3,467	21.34	13.6%	10.0%	2003	10,996	20.79	21.6%	8.6%	−0.06	0.08	0.01
			(3.27)					(3.37)					
Cambodia	2000	6,911	20.58	20.5%	5.9%	2005	7,845	20.90	19.9%	9.2%	0.064	−0.01	0.00
			(2.74)					(3.04)					
Cameroon	1998	1,661	22.90	7.2%	23.4%	2004	4,646	23.57	6.3%	28.5%	0.112	−0.01	0.00
			(3.76)					(4.19)					
Chad	1996	3,709	20.66	21.6%	7.0%	2004	2,952	21.07	21.8%	10.6%	0.051	0.00	0.00
			(2.99)					(3.55)					
Colombia	1995	3,319	24.46	3.7%	40.3%	2010	43,950	25.35	4.8%	47.2%	0.06	0.01	0.00
			(4.00)					(4.96)					
Cote d'Ivoire	1994	2,740	22.97	7.8%	23.1%	1998	3,146	22.09	8.2%	14.1%	−0.22	0.00	0.00
			(4.16)					(3.34)					
Egypt	1995	6,777	26.03	1.9%	49.1%	2008	14,840	28.86	0.7%	76.0%	0.217	−0.01	0.00
			(5.14)					(5.46)					
Ethiopia	2000	13,906	19.95	31.2%	5.2%	2005	6,127	20.36	26.6%	6.4%	0.082	−0.05	−0.01
			(2.91)					(3.11)					
Ghana	1993	1,781	21.77	11.7%	12.7%	2008	4,450	23.35	8.9%	27.8%	0.106	−0.03	0.00
			(3.56)					(4.74)					
Guatemala	1995	5,015	23.84	3.5%	30.6%	1998	2,398	24.67	2.5%	39.6%	0.277	−0.01	0.00
			(3.74)					(4.21)					
Guinea	1999	3,347	21.76	11.7%	12.4%	2005	3,574	21.64	13.7%	13.3%	−0.02	0.02	0.00
			(3.40)					(3.38)					
Haiti	1994	1,902	21.22	18.4%	11.9%	2005	4,935	22.31	14.9%	20.5%	0.098	−0.04	0.00
			(3.43)					(4.28)					
India	1998	76,616	20.61	32.0%	12.3%	2005	113,063	21.01	29.6%	15.3%	0.057	−0.02	0.00
			(3.85)					(4.08)					
Jordan	1997	3,082	27.28	2.3%	61.5%	2007	4,527	28.34	1.3%	69.6%	0.106	−0.01	0.00
			(5.55)					(5.77)					
Kazakhstan	1995	3,538	24.53	8.2%	37.0%	1999	2,218	23.92	7.4%	31.5%	−0.15	−0.01	0.00
			(5.49)					(5.10)					
Kenya	1998	3,294	21.99	11.4%	15.1%	2008	7,692	22.92	12.6%	25.3%	0.094	0.01	0.00
			(3.64)					(4.66)					
Lesotho	2004	3,205	24.91	5.5%	40.6%	2009	3,775	24.92	6.1%	39.7%	0.002	0.01	0.00
			(5.30)					(5.52)					
Madagascar	1997	2,627	20.47	19.4%	4.5%	2008	7,674	20.43	25.5%	6.7%	−0	0.06	0.01
			(2.44)					(3.03)					
Malawi	1992	2,342	21.81	8.5%	11.2%	2010	6,881	22.25	8.8%	15.9%	0.024	0.00	0.00
			(3.07)					(3.48)					
Mali	1995	4,306	21.09	16.2%	8.7%	2006	12,506	22.22	13.0%	18.4%	0.102	−0.03	0.00
			(2.99)					(4.13)					
Morocco	1992	2,890	24.07	3.9%	32.7%	2003	15,941	24.15	7.1%	35.9%	0.007	0.03	0.00
			(4.39)					(4.54)					
Mozambique	1997	3,284	21.73	9.8%	10.4%	2003	10,533	22.28	8.3%	15.9%	0.091	−0.01	0.00
			(3.03)					(3.64)					
Namibia	1992	2,268	22.37	13.6%	19.2%	2006	8,962	23.10	16.5%	27.5%	0.052	0.03	0.00
			(4.27)					(5.50)					
Nepal	1996	3,420	19.94	25.3%	1.8%	2006	10,116	20.58	24.0%	7.9%	0.064	−0.01	0.00
			(2.20)					(3.01)					
Nicaragua	1998	12,258	24.74	4.1%	40.5%	2001	11,936	25.43	3.4%	46.1%	0.229	−0.01	0.00
			(4.63)					(4.94)					
Niger	1998	3,454	20.96	19.4%	9.8%	2006	3,947	21.82	18.6%	17.7%	0.107	−0.01	0.00
			(3.29)					(4.12)					
Nigeria	2003	6,606	22.27	15.0%	19.2%	2008	28,900	22.47	12.6%	20.7%	0.041	−0.02	0.00
			(4.28)					(4.36)					
Peru	1991	5,199	24.57	1.3%	37.7%	2004	25,928	25.43	2.0%	47.8%	0.067	0.01	0.00
			(3.67)					(4.40)					
Rwanda	2000	9,168	22.12	8.9%	13.9%	2005	5,211	21.89	9.9%	12.0%	−0.05	0.01	0.00
			(3.12)					(2.91)					
Tanzania	1996	3,820	21.93	9.4%	13.2%	2004	9,159	22.20	12.4%	17.3%	0.033	0.03	0.00
			(3.31)					(3.90)					
Turkey	1993	2,417	25.91	2.2%	51.1%	2003	3,030	26.60	1.7%	57.5%	0.068	0.00	0.00
			(4.93)					(5.11)					
Uganda	1995	3,234	21.75	9.2%	11.0%	2006	2,519	21.97	12.9%	15.1%	0.019	0.04	0.00
			(3.18)					(3.69)					
Zambia	1996	3,902	21.78	9.6%	11.8%	2007	6,288	22.47	9.6%	18.9%	0.062	0.00	0.00
			(3.08)					(3.94)					
Zimbabwe	1994	1,983	22.91	5.8%	21.2%	2005	8,163	23.05	9.1%	24.8%	0.013	0.03	0.00
			(3.68)					(4.16)					
Total	-	232,150	21.93	19.0%	18.6%	-	465,423	22.95	14.9%	27.0%			
			(4.26)					(4.82)					

Results of the country-level ecological analyses are presented in **table 4**. There were positive but non-significant associations between changes in GDP and FDI and changes in BMI, and no association between change in average tariff rates and change in BMI. There were also positive but non-significant associations between change in GDP and change in both percent overweight and underweight (**tables S1 and S2**). (These results do not change substantially when models are adjusted for baseline values of GDP, FDI, or tariff rates [data not shown]).

**Table 4:** Associations between annual changes in GDP, FDI, and average tariffs and change in BMI in 38 countries.

	Model 1.	Model 2.	Model 3.
	GDP only	FDI and GDP	Average tariffs and GDP
	Association (95% CI)	Association (95% CI)	Association (95% CI)
GDP (in 000 000s)	0.212	0.215	0.212
	(−0.203, 0.626)	(−0.204, 0.633)	(−0.21, 0.634)
FDI (in % GDP)		0.011	
		(−0.093, 0.115)	
Average annual change in tariffs			0.000
			(−0.017, 0.017)
Constant	0.05	0.048	0.05
	(0.01, 0.089)	(0.007, 0.09)	(0.006, 0.093)
			
N	38	38	38
R-squared	0.026	0.026	0.026
			

Associations between of national-level GDP and individual BMI are presented in **table 5**. (Model results including all individual predictors are included as **tables S3 and S4,** and model results excluding data from India are included as **table S5**). In analyses using the full dataset, GDP had a small but positive main association with BMI (0.140 kg/m^2^ increase for every US $1,000 increase in GDP, 95% CI: 0.060, 0.220). However, this result was not seen in either the ecological analysis or in the multilevel analysis excluding data from India: neither of these showed a significant association between GDP and BMI.

**Table 5:** Associations of GDP with BMI and interactive associations of GDP and wealth and GDP and urban residence with BMI.

		GDP	GDP * urban	GDP * wealth
		Adj. Association (95% CI)	Adj. Association (95% CI)	Adj. Association (95% CI)
***Individual-level predictors***				
**Wealth index**				
	Second quintile	0.282	0.311	0.278
		(0.251, 0.313)	(0.280, 0.342)	(0.247, 0.309)
	Third quintile	0.585	0.631	0.579
		(0.552, 0.618)	(0.598, 0.664)	(0.546, 0.612)
	Fourth quintile	1.053	1.083	1.057
		(1.018, 1.088)	(1.048, 1.118)	(1.022, 1.092)
	Highest quintile	2.001	1.992	1.998
		(1.960, 2.042)	(1.951, 2.033)	(1.957, 2.039)
GDP * Wealth index				
	Second quintile			0.028
				(0.012, 0.044)
	Third quintile			0.000
				(−0.016, 0.016)
	Fourth quintile			−0.065
				(−0.083, −0.047)
	Highest quintile			−0.320
				(−0.340, −0.300)
				
***Cluster-level predictors***				
	Urban residence	0.493	0.546	0.456
		(0.458, 0.528)	(0.513, 0.579)	(0.421, 0.491)
	Urban residence * GDP		−0.201	
			(−0.215, −0.187)	
				
***National-level predictors***				
	GDP per capita	0.140	0.291	0.224
		(0.060, 0.220)	(0.211, 0.371)	(0.144, 0.304)
				
***Random effects***				
	Level 1 (Individual)	13.8	13.796	13.787
		(13.753, 13.847)	(13.749, 13.843)	(13.740, 13.834)
	Level 2 (cluster)	0.9	0.875	0.851
		(0.875, 0.925)	(0.851, 0.899)	(0.827, 0.875)
	Level 3 (region)	0.302	0.283	0.285
		(0.255, 0.349)	(0.240, 0.326)	(0.242, 0.328)
	Level 4 (country)	2.959	2.716	2.941
		(1.611, 4.307)	(1.479, 3.953)	(1.602, 4.280)
				
Constant		19.358	19.52	19.353
		(18.741, 19.975)	(18.924, 20.116)	(18.740, 19.966)
				
N		697573	697573	697573
				

Model also adjusted for age (5-year groups), educational attainment (no/incomplete primary, complete primary/incomplete secondary, complete secondary and higher), marital status, and survey year (categorical).

**Figure 1:**
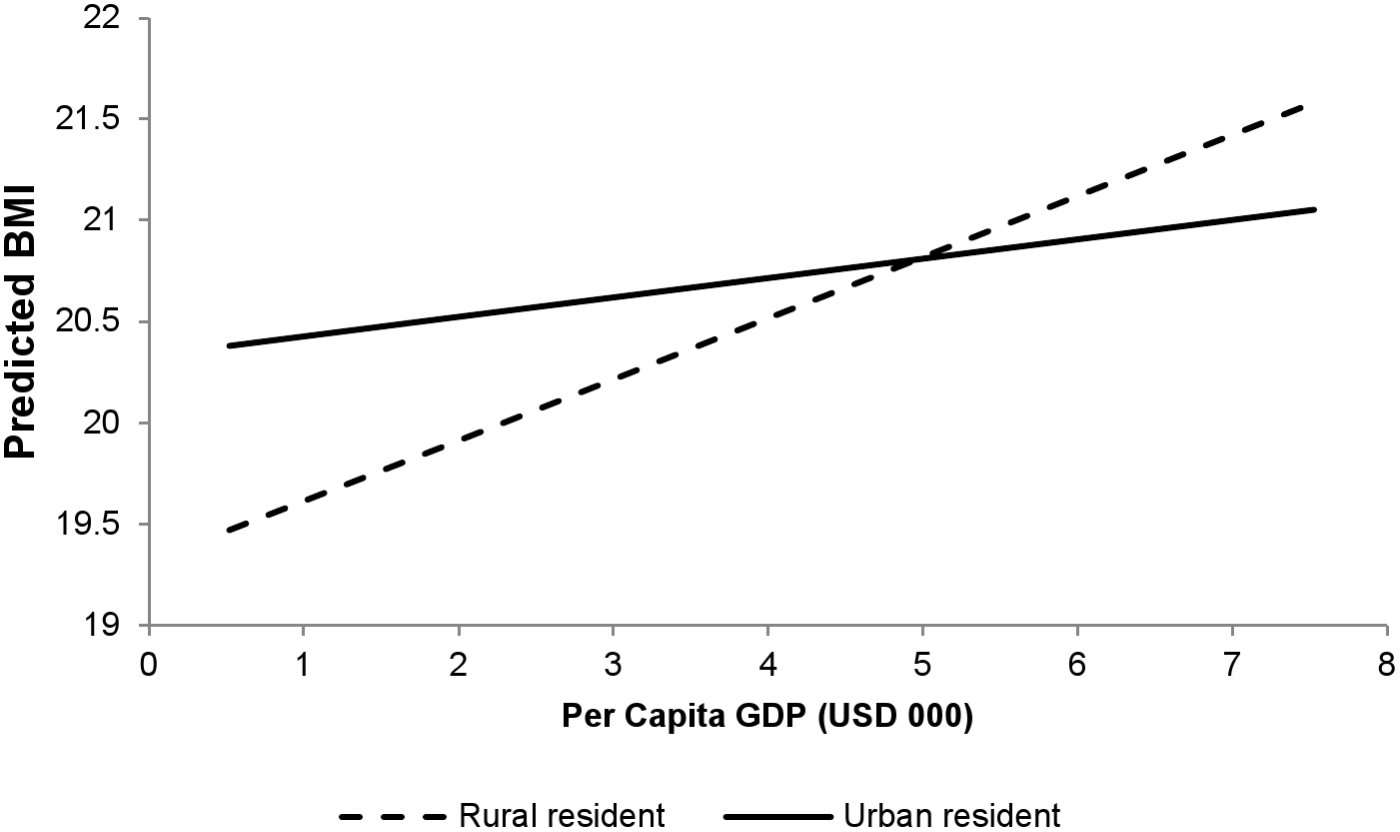
Per capita GDP and predicted BMI by type of residence.

**Figure 2:**
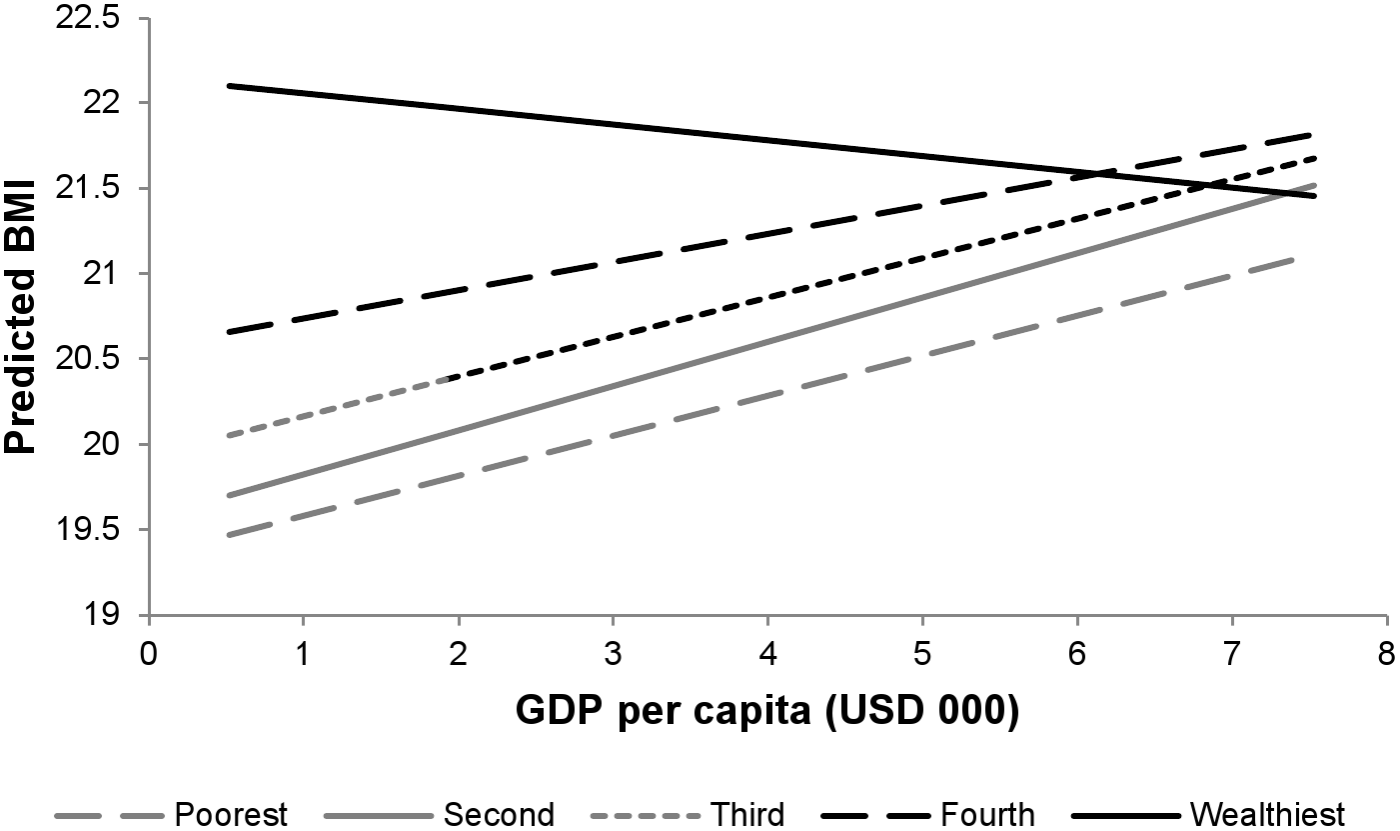
Per capita GDP and predicted BMI by wealth group.

Both urban residence and wealth had significant and positive associations with BMI (urban association: 0.493 kg/m^2^, 95% CI: 0.458, 0.528; association with highest wealth group compared to lowest: 2.001 kg/m^2^, 95% CI: 1.960, 2.042). The positive association of GDP with BMI was much smaller among urban residents (estimated association for rural residents: 0.291 kg/m^2^, 95% CI: 0.211, 0.371; differential association for urban residents: −0.201, 95% CI: −0.215, −0.187) (**figure 1**), and was negative among the wealthiest residents (estimated differential association for the wealthiest respondents: −0.320 kg/m^2^, 95% CI: −0.340, −0.300) (**figure 2**). Results of the secondary analysis with over- and underweight as outcomes show similar patterns, with overweight positively and underweight negatively associated with national GDP, urban residence, and wealth (**table S6**). However, there was a small but positive differential association of GDP per capita on underweight among urban residents, suggesting GDP increases may have less of a beneficial impact on under nutrition among urban populations (adjusted odds ratio for GDP per capita among rural residents: 0.906, 95% CI: 0.890, 0.922; adjusted differential odds ratio for urban residents: 1.030, 95% CI: 1.020, 1.041).

**Figure 3:**
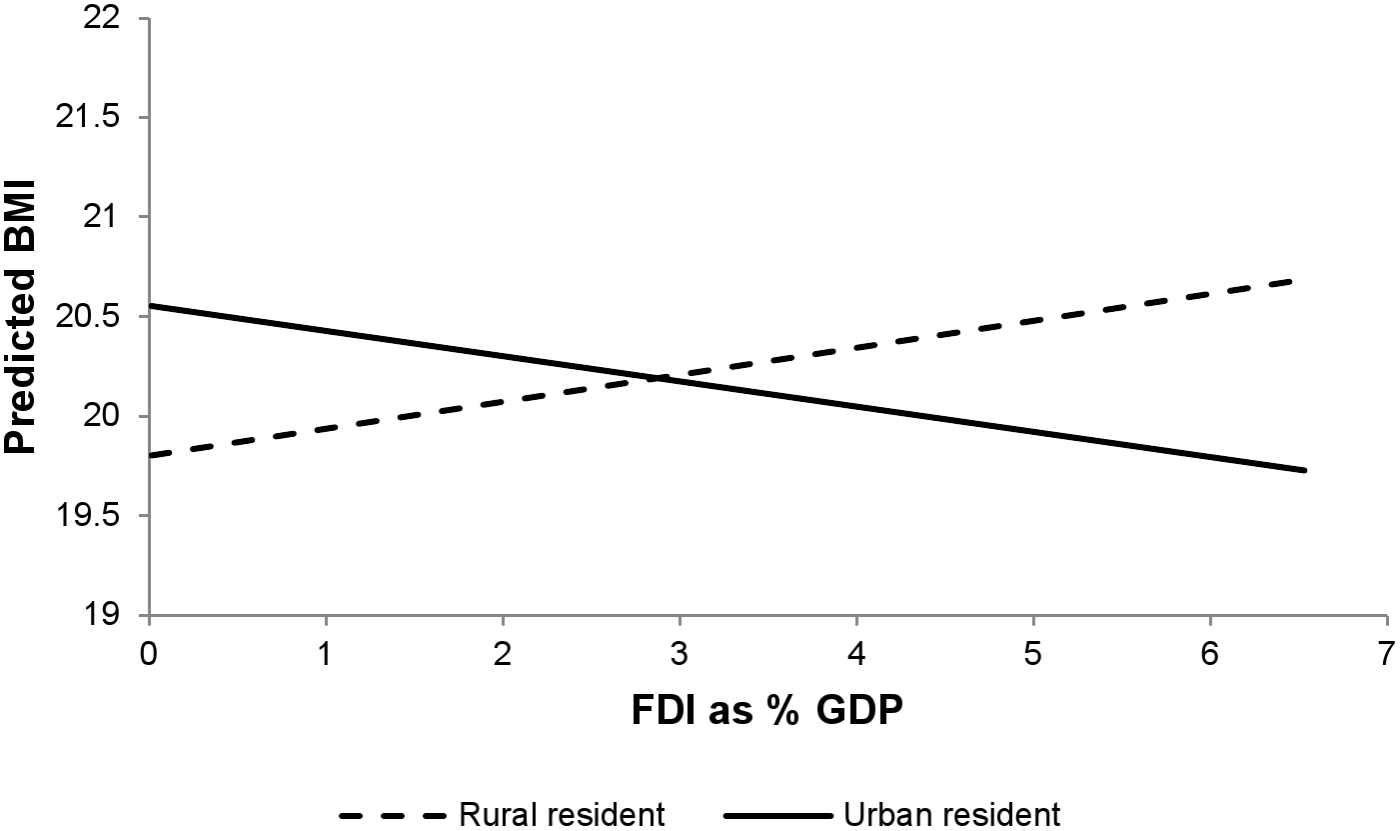
FDI and predicted BMI by type of residence.

**Figure 4:**
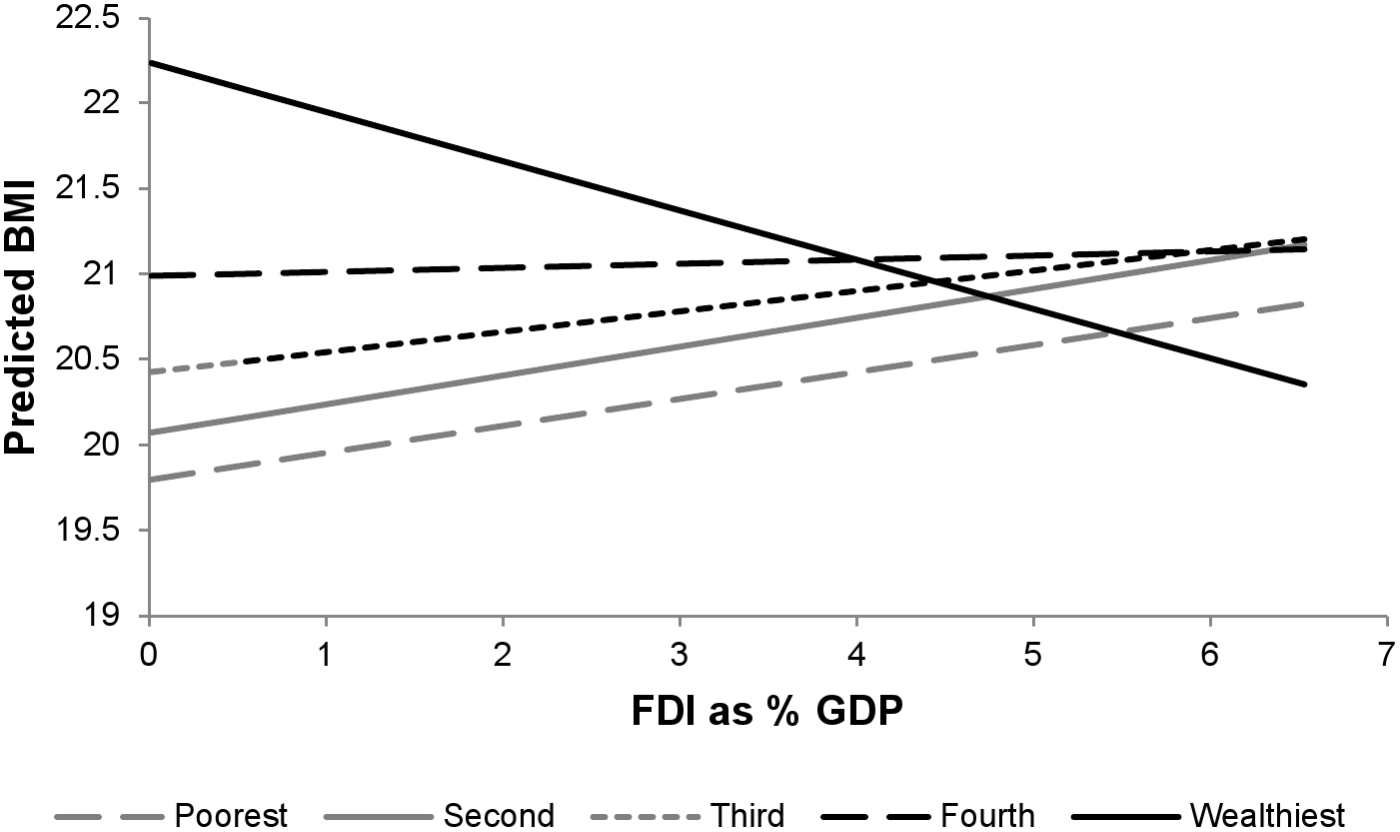
FDI and predicted BMI by wealth group.

Associations of FDI as percent GDP with BMI are presented in **table 6**. As expected given the results of the ecological analysis above, there was no main association of FDI on BMI after adjustment for GDP (estimated effect of one percentage point change in FDI/GDP: 0.000 kg/m^2^, 95% CI: −0.053, 0.053). However, FDI did have significant differential associations in rural and urban areas, with stronger positive associations of FDI and BMI seen in rural areas (main association of FDI in rural areas: 0.129 kg/m^2^, 95% CI: 0.074, 0.184, differential associations in urban areas compared with rural areas: −0.252 kg/m^2^, 95% CI: −0.277, −0.227) (**figure 3**). FDI was negatively associated with BMI among the wealthiest respondents and positively associated among lower wealth groups (main association of FDI among poorest respondents: 0.149 kg/m^2^, 95% CI: 0.092, 0.206; differential association among wealthiest compared with poorest: −0.436 kg/m^2^, 95% CI: −0.467, −0.405). (**figure 4**). The association of FDI with overweight was also small among the wealthiest compared to the poorest (adjusted odds ratio of FDI among poorest: 1.235, 95% CI: 1.223, 1.247; differential association among wealthiest: 0.745, 95% CI: 0.732, 0.758) (**table S7**).

**Table 6:** Association of FDI with BMI and interactive associations of FDI and wealth and FDI and urban residence with BMI.

		FDI	FDI * urban	FDI * wealth
		Adj. association (95% CI)	Adj. association (95% CI)	Adj. association (95% CI)
***Individual-level predictors***				
**Wealth index**				
	Second quintile	0.282	0.293	0.285
		(0.251, 0.313)	(0.262, 0.324)	(0.254, 0.316)
	Third quintile	0.585	0.602	0.591
		(0.552, 0.618)	(0.569, 0.635)	(0.558, 0.624)
	Fourth quintile	1.053	1.066	1.056
		(1.018, 1.088)	(1.031, 1.101)	(1.021, 1.091)
	Highest quintile	2.001	2.001	1.974
		(1.960, 2.042)	(1.960, 2.042)	(1.933, 2.015)
				
FDI * Wealth index				
	Second quintile			0.015
				(−0.012, 0.042)
	Third quintile			−0.033
				(−0.062, −0.004)
	Fourth quintile			−0.126
				(−0.155, −0.097)
	Highest quintile			−0.436
				(−0.467, −0.405)
				
***Cluster-level predictors***				
	Urban residence	0.493	0.514	0.50
		(0.458, 0.528)	(0.481, 0.547)	(0.467, 0.533)
	Urban residence * FDI		−0.252	
			(−0.277, −0.227)	
				
***National-level predictors***				
	GDP per capita	0.140	0.175	0.135
		(0.060, 0.220)	(0.095, 0.255)	(0.055, 0.215)
	FDI(%GDP)	0.000	0.129	0.149
		(−0.053, 0.053)	(0.074, 0.184)	(0.092, 0.206)
				
***Random effects***				
	Level 1 (Individual)	13.8	13.8	13.792
		(13.753, 13.847)	(13.753, 13.847)	(13.745, 13.839)
	Level 2 (cluster)	0.9	0.882	0.87
		(0.875, 0.925)	(0.857, 0.907)	(0.846, 0.894)
	Level 3 (region)	0.302	0.309	0.315
		(0.255, 0.349)	(0.262, 0.356)	(0.266, 0.364)
	Level 4 (country)	2.959	2.838	2.902
		(1.611, 4.307)	(1.542, 4.134)	(1.581, 4.223)
				
Constant		19.359	19.39	19.406
		(18.736, 19.982)	(18.777, 20.003)	(18.787, 20.025)
				
**N**		697573	697573	697573

Model also adjusted for age (5-year groups), educational attainment (no/incomplete primary, complete primary/incomplete secondary, complete secondary and higher), marital status, and survey year (categorical).

**Figure 5:**
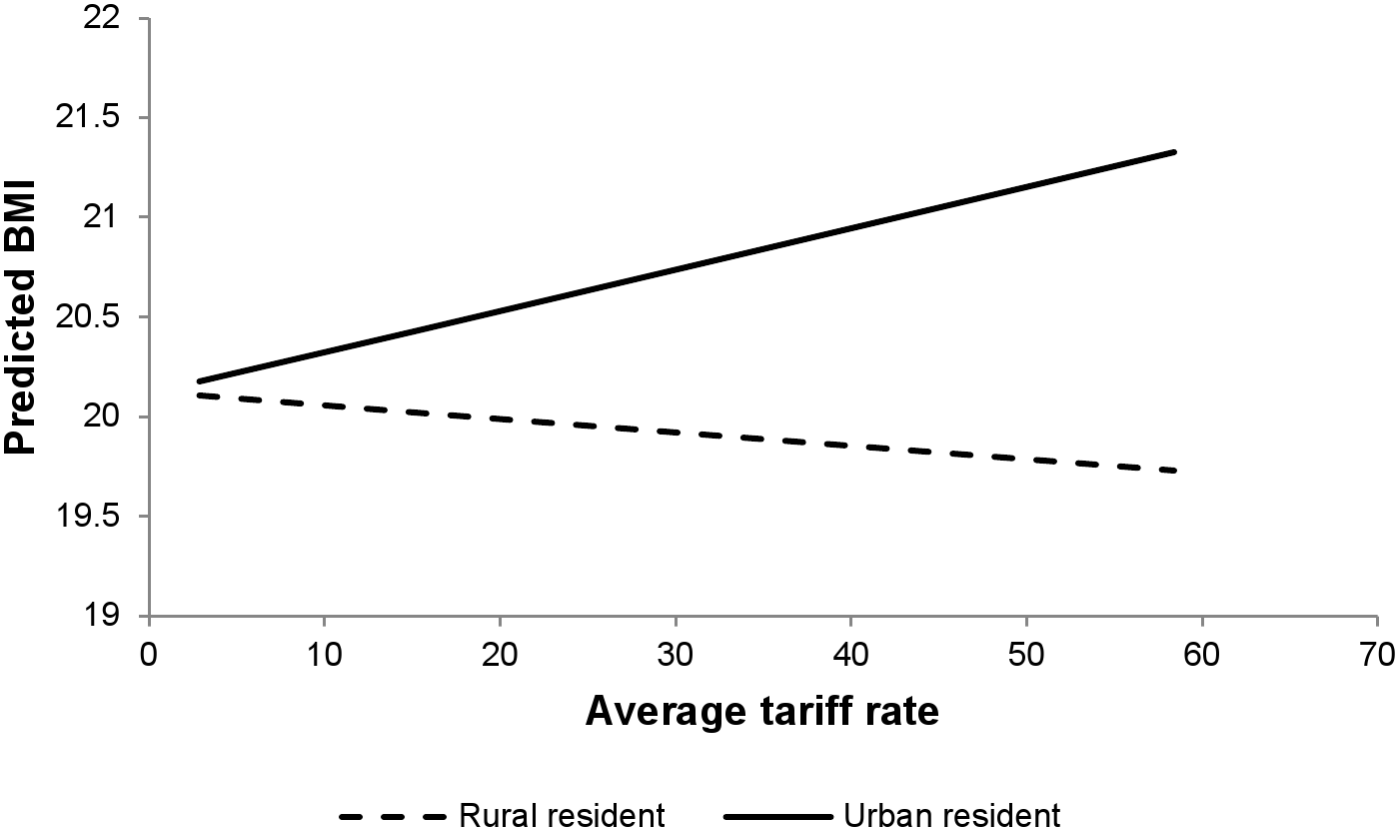
Average tariff rate and predicted BMI by type of residence.

**Figure 6:**
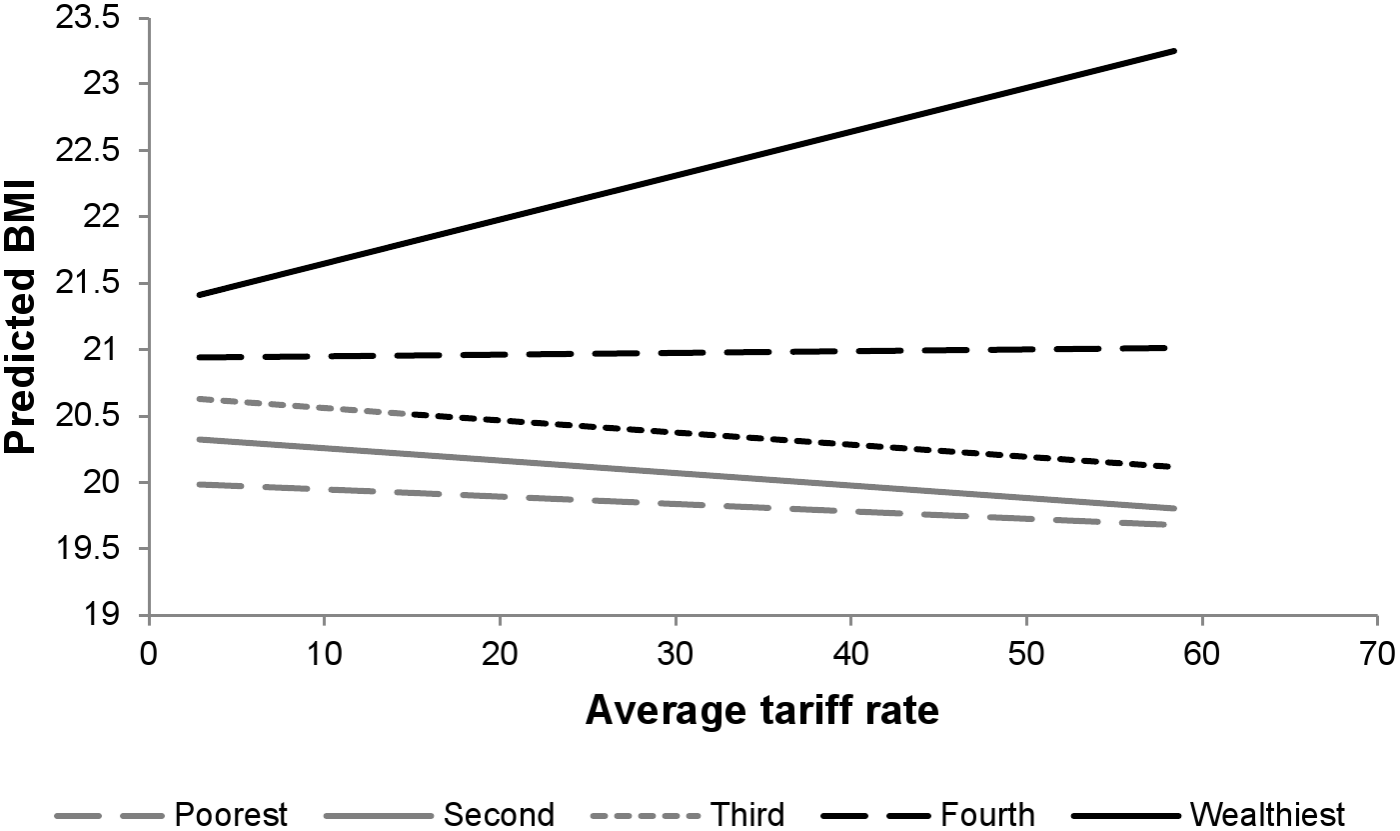
Average tariff rate and predicted BMI by wealth group.

Associations between average tariff rates and individual BMI are presented in **table 7**. While there was no significant main association of average tariff rate on BMI (estimated association with one percentage point change in tariff rate: 0.003 kg/m^2^, 95% CI: −0.001, 0.007), there were significant interactions between both urban residence and tariff rate and SES and tariff rate. Tariff rates had a stronger positive association among urban residents (estimated association of tariff rates on BMI among rural residents: −0.005 kg/m^2^, 95% CI: −0.009, −0.001; differential association among urban residents: 0.026 kg/m^2^, 95% CI: 0.024, 0.028) (**figure 5**) and among the wealthy (estimated association among the poorest: −0.004 kg/m^2^, 95% CI: −0.008, 0.000; differential association among the wealthiest: 0.036 kg/m^2^, 95% CI: 0.032, 0.040) (**figure 6**). This negative association of tariff on BMI translates into a positive correlation between trade openness and BMI (adjusted odds ratio: 0.036, 95% CI: 1.036, 1.036), suggesting that those who live in countries with high tariffs may be somewhat more prone to under nutrition. (**table S8** presents associations between tariffs and over- and underweight.).

**Table 7:** Associations between tariff rates and BMI and interactive associations of tariff rates and wealth and tariff rates and urban residence with BMI.

		Average tariff	Average tariff * urban	Average tariff * wealth
		Adj. association (95% CI)	Adj. association (95% CI)	Adj. association (95% CI)
***Individual-level predictors***				
**Wealth index**				
	Second quintile	0.282	0.287	0.282
		(0.251, 0.313)	(0.256, 0.318)	(0.251, 0.313)
	Third quintile	0.585	0.595	0.586
		(0.552, 0.618)	(0.562, 0.628)	(0.553, 0.619)
	Fourth quintile	1.053	1.061	1.057
		(1.018, 1.088)	(1.026, 1.096)	(1.022, 1.092)
	Highest quintile	2.001	2.001	1.997
		(1.960, 2.042)	(1.960, 2.042)	(1.956, 2.038)
				
FDI * Wealth index				
	Second quintile			−0.004
				(−0.008, 0.000)
	Third quintile			−0.005
				(−0.009, −0.001)
	Fourth quintile			0.005
				(0.001, 0.009)
	Highest quintile			0.036
				(0.032, 0.040)
				
***Cluster-level predictors***				
	Urban residence	0.493	0.511	0.495
		(0.458, 0.528)	(0.478, 0.544)	(0.462, 0.528)
	Urban residence * Tariff rates		0.026	
			(0.024, 0.028)	
				
***National-level predictors***				
	GDP per capita	0.129	0.182	0.125
		(0.049, 0.209)	(0.102, 0.262)	(0.045, 0.205)
	Average tariff (%)	0.003	−0.005	−0.004
		(−0.001, 0.007)	(−0.009, −0.001)	(−0.008, 0.000)
				
***Random effects***				
	Level 1 (Individual)	13.8	13.8	13.792
		(13.753, 13.847)	(13.753, 13.847)	(13.745, 13.839)
	Level 2 (cluster)	0.9	0.887	0.881
		(0.875, 0.925)	(0.862, 0.912)	(0.856, 0.906)
	Level 3 (region)	0.302	0.306	0.307
		(0.255, 0.349)	(0.259, 0.353)	(0.260, 0.354)
	Level 4 (country)	3.032	2.828	3.061
		(1.652, 4.412)	(1.538, 4.118)	(1.669, 4.453)
				
Constant		19.326	19.424	19.325
		(18.701, 19.951)	(18.816, 20.032)	(18.698, 19.952)
				
**N**		697573	697573	697573

## Discussion

The ecological analyses presented above indicate that there were no significant associations between change in economic factors and change in BMI or prevalence of overweight at the national level. However, multilevel analyses found that individual wealth and place of residence modify the associations between these national-level indicators and BMI, underscoring the need to measure how national trends affect both mean changes in health indicators and how they increase or decrease health disparities.

These analyses also suggest that, among rural and poor women in the countries included in this study, increasing GDP is associated with increased mean BMI, decreased odds of underweight, and increased odds of overweight. In the poorest countries, these women tend to have lower body weight than their urban and wealthy counterparts in lower income countries [17], [34], [35], [40], [67]. However, in middle income countries both within and outside this study, such as Mexico and Egypt, prevalence of overweight and obesity in rural areas is substantial, and is quickly catching up to prevalence of overweight and obesity in urban areas [68], [69].

Both measures of economic openness tended to have the same result: increased openness, whether measured through increases in foreign investment or decreases in average tariff rates, tend to have strong associations with the BMI of the wealthiest respondents or respondents living in urban areas. This finding confirms that those who are expected by globalization to have the most access to global markets in developing countries – wealthy urban residents – see the strongest associations between trade openness and BMI. This also suggests that, as openness to trade increases, the variability in BMI across the population may also increase, leading to higher rates of both under- and overweight, rather than a general shift of the population distribution of BMI away from underweight.

The inverse association between FDI and BMI among only the wealthiest respondents is notable, and does not fit with the general trend of positive associations between indicators of development or trade openness and BMI. This finding may suggest that increased foreign presence leads to shifts in perceptions of obesity. One possible mechanism underlying this shift is changing attitudes toward obesity among the wealthiest in places with increased exposure to foreign cultural influences. Studies have found that high body mass is generally positively perceived among women in Africa [2], [70], [71], though not in India [72], and the literature on globalization and obesity argues that Western cultural norms about obesity and body weight will also become more prevalent in developing countries as a result of globalization [9], [10]. High-SES and urban women may exhibit these changes first because they have increased exposure to media and marketing.

This study has several limitations and strengths. First, the global scope of this study serves as both a strength and a weakness: while, on the one hand, the breadth of the population included allows for the identification of broad international trends in mean BMI, it at the same time makes it difficult to pinpoint specific mechanisms, such as behavioral trends, policy changes, or environmental modifications, linking trade or development and BMI. Particularly missing from this study is information collected at the city or regional level; while our study includes individual and national data, regional economic development trends and the local built environment are likely to affect nutritional status as well. Studies including more detailed economic data from smaller geographic areas, data collected over a longer time span, and longitudinal data on both women and men could provide more nuanced information on the roles of trade and economic development in shaping individual health over time.

This analysis uses recent data from Demographic and Health Surveys, which are primarily conducted in lower and lower-middle income countries. Consequently, the geographic scope of the analysis is somewhat limited, and several middle-income countries that frequently appear in the literature on chronic disease, including China, Mexico, and Brazil, are not included in this analysis. This limits the generalizability of these findings primarily to lower-income countries, where rates of obesity and overweight are relatively low. The surveys used in this analysis were conducted at different times across countries, and the measures of urban residence and overall wealth are country-specific. In this analysis, urban residence is defined by each country, and these definitions vary widely within regions [73]–[75]. The definition of urban residence used is dichotomous; while this is the most commonly used type of definition for most applications and will be most familiar to both researchers and policymakers, several authors have suggested that an urban gradient would be a more informative measure for understanding the effects of type of residence on health [73], [76]. The wealth index measure used as a proxy for SES was designed to be comparable across countries [63], but does not account for changes in national wealth over time. Finally, this analysis does not include measures of SES that would be particularly relevant for assessing the effect of SES and employment type on BMI, particularly in rural areas. The wealth measure is an imperfect proxy for SES in rural areas because these measures incorporate housing infrastructure that is more likely to be found in urban areas [62], [77]. Specific data on the type (agricultural, non-agricultural) and nature (sedentary, active) of respondents’ employment, which could affect BMI by reducing physical activity or increasing income, are not included in this dataset. Finally, because the individual-level data used only includes information collected from women, the role of gender in modifying the effects of economic development on health cannot be investigated here [78].

There are also limitations to the data on GDP and FDI used. The data on FDI is not fully comparable across countries, because of national discrepancies in the definition of FDI, methods of collecting FDI data, and accounting and valuation practices [79]. Because the trade data assembled and disseminated by the FAO is collected by national agencies, there are likely to be differences in the quality of the data collected due to disparities in local data collection capacity and resources [80]. Additionally, the macroeconomic indicators proposed for this analysis are relatively crude markers of national trade and food environments, and incorporating only six years of GDP data may be too short a span to identify associations between these indicators and individual body weight. Finally, the availability of data on tariff rates was limited, particularly for years prior to 2000; for this reason, an imputed estimate of tariffs for the year of survey only was used. Because data on tariffs prior to the survey year were not incorporated into the analysis, these results do not account for the potential lagged effect of tariff reductions on body weight.

Finally, while the ecological analyses failed to identify significant associations between national change in economic indicators and mean body weight, this may be due to the small number of countries and short timeframe included in the dataset. However, recent models of adult metabolism have suggested that weight change as a result of changes in caloric intake happen relatively quickly, with half of body weight increase occurring within one year and 95% within three years [81], making the 3–18 year spans between surveys included in the analysis a valid time period in which to assess population changes in body weight.

To summarize, this analysis does not identify large or statistically significant associations between changes in economic development indicators and mean changes in BMI. However, there appear to be marked differentials in how these indicators are associated with BMI among individuals within populations of lower-income countries. While GDP and FDI tended to be positively associated with BMI among poor, rural individuals, increasing tariffs tended to be associated with increases in overweight among the wealthiest, and also led to small increases in underweight. These divergent findings underscore the complexity of the effects of development on health, the variety of mechanisms through which cultural change may effect body weight, and the importance of considering how the health effects of “globalizing” economic and cultural trends are modified by individual-level wealth and residence.

## Supporting Information

Table S1 **Annual change in GDP, FDI, and average tariffs by annual change prevalence of overweight for 38 countries.**
(DOCX)

Table S2 **Annual change in GDP, FDI, and average tariffs by annual change in prevalence of underweight for 38 countries.**
(DOCX)

Table S3 **Associations of age, educational attainment, wealth, marital status, urban residence, and national GDP with BMI.**
(DOCX)

Table S4 **Odds ratios comparing underweight and normal weight and overweight and normal weight respondents by age, educational attainment, wealth index, marital status, urban residence, and national GDP.**
(DOCX)

Table S5 **Associations of GDP with BMI and interactive associations of GDP and wealth and GDP and urban residence with BMI, 37 countries (India excluded).**
(DOCX)

Table S6 **Odds ratios comparing underweight and normal weight and overweight and normal weight respondents by GDP and wealth and GDP and urban residence.**
(DOCX)

Table S7 **Odds ratios comparing underweight and normal weight and overweight and normal weight respondents by FDI and wealth and FDI and urban residence.**
(DOCX)

Table S8 **Odds ratios comparing underweight and normal weight and overweight and normal weight respondents by tariff rate and wealth and tariff rate and urban residence.**
(DOCX)
