# Supplementary material for: National Economic Development and Disparities in Body Mass Index: A Cross-Sectional Study of Data from 38 Countries
Source: PLoS One. 2014 Jun 11;9(6):e99327. doi: 10.1371/journal.pone.0099327 (PMC4053361; doi:10.1371/journal.pone.0099327)
Supplement: Table S1 — Annual change in GDP, FDI, and average tariffs by annual change prevalence of overweight for 38 countries. (DOCX) [file pone.0099327.s001.docx]

**Table S1. Annual change in GDP, FDI, and average tariffs by annual change prevalence of overweight for 38 countries**

|  | **Model 1. GDP only** | **Model 2. FDI and GDP** | **Model 3. Average tariffs and GDP** |
| --- | --- | --- | --- |
|  | **Effect (95% CI)** | **Effect (95% CI)** | **Effect (95% CI)** |
| GDP (in 000 000s) | 1.019 | 1.019 | 1.019 |
|  | (0.979, 1.061) | (0.978, 1.061) | (0.978, 1.061) |
| FDI (in % GDP) |  | 1.000 |  |
|  |  | (0.991, 1.008) |  |
| Average annual change in tariffs |  |  | 1.000 |
|  |  |  | (0.999, 1.001) |
| Constant | 1.005 | 1.005 | 1.005 |
|  | (1.001, 1.008) | (1.001, 1.008) | (1.001, 1.009) |
|  |  |  |  |
| N | 38 | 38 | 38 |
| R-squared | 0.024 | 0.024 | 0.025 |
|  |  |  |  |
