# Supplementary material for: National Economic Development and Disparities in Body Mass Index: A Cross-Sectional Study of Data from 38 Countries
Source: PLoS One. 2014 Jun 11;9(6):e99327. doi: 10.1371/journal.pone.0099327 (PMC4053361; doi:10.1371/journal.pone.0099327)
Supplement: Table S2 — Annual change in GDP, FDI, and average tariffs by annual change in prevalence of underweight for 38 countries. (DOCX) [file pone.0099327.s002.docx]

**Table S2. Annual change in GDP, FDI, and average tariffs by annual change in prevalence of underweight for 38 countries**

|  | **Model 1. GDP only** | **Model 2. FDI and GDP** | **Model 3. Average tariffs and GDP** |
| --- | --- | --- | --- |
|  | **Effect (95% CI)** | **Effect (95% CI)** | **Effect (95% CI)** |
| GDP (in 000 000s) | 1.005 | 1.006 | 1.004 |
|  | (0.994, 1.016) | (0.994, 1.017) | (0.993, 1.015) |
| FDI (in % GDP) |  | 1.001 |  |
|  |  | (0.997, 1.006) |  |
| Average annual change in tariffs |  |  | 1.001 |
|  |  |  | (0.999, 1.003) |
| Constant | 0.999 | 0.999 | 1 |
|  | (0.997, 1.001) | (0.997, 1.001) | (0.998, 1.001) |
|  |  |  |  |
| N | 38 | 38 | 38 |
| R-squared | 0.006 | 0.009 | 0.044 |
