# Supplementary material for: National Economic Development and Disparities in Body Mass Index: A Cross-Sectional Study of Data from 38 Countries
Source: PLoS One. 2014 Jun 11;9(6):e99327. doi: 10.1371/journal.pone.0099327 (PMC4053361; doi:10.1371/journal.pone.0099327)
Supplement: Table S3 — Associations of age, educational attainment, wealth, marital status, urban residence, and national GDP with BMI. (DOCX) [file pone.0099327.s003.docx]

**Table S3. Associations of age, educational attainment, wealth, marital status, urban residence, and national GDP with BMI**

|  |  | **Adj. Effect** |
| --- | --- | --- |
|  |  | **(SE)** |
| ***Individual-level predictors*** | |  |
| **Age (years)** | |  |
|  | 20-24 | 0.567 |
|  |  | (0.536, 0.598) |
|  | 25-29 | 1.191 |
|  |  | (1.158, 1.224) |
|  | 30-34 | 1.845 |
|  |  | (1.810, 1.880) |
|  | 35-39 | 2.318 |
|  |  | (2.281, 2.355) |
|  | 40-44 | 2.704 |
|  |  | (2.665, 2.743) |
|  | 45-49 | 2.935 |
|  |  | (2.894, 2.976) |
| **Educational attainment** | |  |
|  | Complete primary/incomplete secondary | 0.362 |
|  |  | (0.337, 0.387) |
|  | Complete secondary and higher | 0.187 |
|  |  | (0.154, 0.220) |
| **Wealth index** | |  |
|  | Second quintile | 0.282 |
|  |  | (0.251, 0.313) |
|  | Third quintile | 0.585 |
|  |  | (0.552, 0.618) |
|  | Fourth quintile | 1.053 |
|  |  | (1.018, 1.088) |
|  | Highest quintile | 2.001 |
|  |  | (1.960, 2.042) |
| **Marital status** | |  |
|  | Ever married | 0.859 |
|  |  | (0.832, 0.886) |
| ***Cluster-level predictors*** | |  |
|  | Urban residence | 0.493 |
|  |  | (0.458, 0.528) |
| ***National-level predictors*** | |  |
|  | GDP per capita (000s) | 0.140 |
|  |  | (0.060, 0.220) |
| **Random effects** | |  |
|  | Level 1 (Individual) | 13.800 |
|  |  | (13.753, 13.847) |
|  | Level 2 (cluster) | 0.900 |
|  |  | (0.875, 0.925) |
|  | Level 3 (region) | 0.302 |
|  |  | (0.255, 0.349) |
|  | Level 4 (country) | 2.959 |
|  |  | (1.611, 4.307) |
|  |  |  |
| **Constant** |  | 19.358 |
|  |  | (18.741, 19.975) |
|  |  |  |
| **N** |  | 697573 |
