# Supplementary material for: National Economic Development and Disparities in Body Mass Index: A Cross-Sectional Study of Data from 38 Countries
Source: PLoS One. 2014 Jun 11;9(6):e99327. doi: 10.1371/journal.pone.0099327 (PMC4053361; doi:10.1371/journal.pone.0099327)
Supplement: Table S4 — Odds ratios comparing underweight and normal weight and overweight and normal weight respondents by age, educational attainment, wealth index, marital status, urban residence, and national GDP. (DOCX) [file pone.0099327.s004.docx]

**Table S4. Odds ratios comparing underweight and normal weight and overweight and normal weight respondents by age, educational attainment, wealth index, marital status, urban residence, and national GDP**

|  |  | **OR** |
| --- | --- | --- |
|  |  | **(95% CI)** |
| **Underweight** | |  |
| ***Individual-level predictors*** | |  |
| **Age** |  |  |
|  | 20-24 | 0.763 |
|  |  | (0.747, 0.780) |
|  | 25-29 | 0.705 |
|  |  | (0.689, 0.722) |
|  | 30-34 | 0.666 |
|  |  | (0.650, 0.684) |
|  | 35-39 | 0.653 |
|  |  | (0.635, 0.671) |
|  | 40-44 | 0.664 |
|  |  | (0.645, 0.684) |
|  | 45-49 | 0.698 |
|  |  | (0.676, 0.720) |
| **Educational attainment** | |  |
|  | Complete primary/incomplete secondary | 0.826 |
|  |  | (0.812, 0.841) |
|  | Complete secondary and higher | 0.656 |
|  |  | (0.640, 0.673) |
| **Wealth index** | |  |
|  | Second quintile | 0.938 |
|  |  | (0.918, 0.958) |
|  | Third quintile | 0.882 |
|  |  | (0.864, 0.902) |
|  | Fourth quintile | 0.775 |
|  |  | (0.757, 0.793) |
|  | Highest quintile | 0.628 |
|  |  | (0.611, 0.646) |
|  |  |  |
| **Ever-married** | | 0.925 |
|  |  | (0.907, 0.943) |
|  |  |  |
| ***Cluster-level predictors*** | |  |
|  | Urban residence | 0.897 |
|  |  | (0.881, 0.913) |
|  |  |  |
| ***National-level predictors*** | |  |
|  | GDP per capita | 0.946 |
|  |  | (0.940, 0.951) |
|  |  |  |
| **Constant** | | 0.050 |
|  |  | (0.039, 0.063) |
| **Overweight** | |  |
| ***Individual-level predictors*** | |  |
| **Age** |  |  |
|  | 20-24 | 1.611 |
|  |  | (1.568, 1.656) |
|  | 25-29 | 2.573 |
|  |  | (2.503, 2.644) |
|  | 30-34 | 3.728 |
|  |  | (3.628, 3.832) |
|  | 35-39 | 4.735 |
|  |  | (4.598, 4.876) |
|  | 40-44 | 5.714 |
|  |  | (5.549, 5.885) |
|  | 45-49 | 6.373 |
|  |  | (6.176, 6.576) |
| **Educational attainment** | |  |
|  | Complete primary/incomplete secondary | 1.376 |
|  |  | (1.354, 1.397) |
|  | Complete secondary and higher | 1.228 |
|  |  | (1.204, 1.252) |
| **Wealth index** | |  |
|  | Second quintile | 1.188 |
|  |  | (0.977, 1.216) |
|  | Third quintile | 1.313 |
|  |  | (1.282, 1.344) |
|  | Fourth quintile | 1.605 |
|  |  | (1.567, 1.643) |
|  | Highest quintile | 2.413 |
|  |  | (2.353, 2.476) |
|  |  |  |
| **Ever-married** | | 0.925 |
|  |  | (0.907, 0.943) |
|  |  |  |
| ***Cluster-level predictors*** | |  |
|  | Urban residence | 1.505 |
|  |  | (1.482, 1.529) |
|  |  |  |
| ***National-level predictors*** | |  |
|  | GDP per capita | 1.397 |
|  |  | (1.391, 1.402) |
|  |  |  |
| **Constant** | | 0.044 |
|  |  | (0.042, 0.047) |
|  |  |  |
| **N** |  | 697573 |
