# Supplementary material for: National Economic Development and Disparities in Body Mass Index: A Cross-Sectional Study of Data from 38 Countries
Source: PLoS One. 2014 Jun 11;9(6):e99327. doi: 10.1371/journal.pone.0099327 (PMC4053361; doi:10.1371/journal.pone.0099327)
Supplement: Table S5 — Associations of GDP with BMI and interactive associations of GDP and wealth and GDP and urban residence with BMI, 37 countries (India excluded). (DOCX) [file pone.0099327.s005.docx]

**Table S5. Associations of GDP with BMI and interactive associations of GDP and wealth and GDP and urban residence with BMI, 37 countries (India excluded)**

|  |  | **GDP** | **GDP * urban** | **GDP * wealth** | **FDI** | **FDI * urban** | **FDI * wealth** | **Tariff rates** | **Tariff rates * urban** | **Tariff rates * wealth** |
| --- | --- | --- | --- | --- | --- | --- | --- | --- | --- | --- |
|  |  | Adj. association (95% CI) | Adj. association (95% CI) | Adj. association (95% CI) | Adj. association (95% CI) | Adj. association (95% CI) | Adj. association (95% CI) | Adj. association (95% CI) | Adj. association (95% CI) | Adj. association (95% CI) |
| ***Individual-level predictors*** | |  |  |  |  |  |  |  |  |  |
| **Wealth index** | |  |  |  |  |  |  |  |  |  |
|  | Second quintile | 0.322 | 0.317 | 0.354 | 0.322 | 0.326 | 0.321 | 0.322 | 0.322 | 0.322 |
|  |  | (0.285, 0.359) | (0.280, 0.354) | (0.317, 0.391) | (0.285, 0.359) | (0.289, 0.363) | (0.284, 0.358) | (0.285, 0.359) | (0.285, 0.359) | (0.285, 0.359) |
|  | Third quintile | 0.623 | 0.616 | 0.677 | 0.623 | 0.628 | 0.621 | 0.622 | 0.624 | 0.622 |
|  |  | (0.584, 0.662) | (0.577, 0.655) | (0.638, 0.716) | (0.584, 0.662) | (0.589, 0.667) | (0.582, 0.660) | (0.583, 0.661) | (0.585, 0.663) | (0.583, 0.661) |
|  | Fourth quintile | 0.993 | 0.998 | 1.026 | 0.993 | 0.996 | 0.988 | 0.993 | 0.993 | 0.992 |
|  |  | (0.950, 1.036) | (0.955, 1.041) | (0.983, 1.069) | (0.950, 1.036) | (0.953, 1.039) | (0.945, 1.031) | (0.950, 1.036) | (0.950, 1.036) | (0.949, 1.035) |
|  | Highest quintile | 1.663 | 1.660 | 1.643 | 1.663 | 1.658 | 1.652 | 1.662 | 1.659 | 1.661 |
|  |  | (1.614, 1.712) | (1.611, 1.709) | (1.594, 1.692) | (1.614, 1.712) | (1.609, 1.707) | (1.603, 1.701) | (1.613, 1.711) | (1.610, 1.708) | (1.612, 1.710) |
| **GDP * Wealth index** | |  |  |  |  |  |  |  |  |  |
|  | Second quintile |  |  | 0.031 |  |  |  |  |  |  |
|  |  |  |  | (0.015, 0.047) |  |  |  |  |  |  |
|  | Third quintile |  |  | 0.004 |  |  |  |  |  |  |
|  |  |  |  | (-0.014, 0.022) |  |  |  |  |  |  |
|  | Fourth quintile |  |  | -0.051 |  |  |  |  |  |  |
|  |  |  |  | (-0.069, -0.033) |  |  |  |  |  |  |
|  | Highest quintile |  |  | -0.289 |  |  |  |  |  |  |
|  |  |  |  | (-0.309, -0.269) |  |  |  |  |  |  |
| **FDI * Wealth index** | |  |  |  |  |  |  |  |  |  |
|  | Second quintile |  |  |  |  |  | 0.010 |  |  |  |
|  |  |  |  |  |  |  | (-0.021, 0.041) |  |  |  |
|  | Third quintile |  |  |  |  |  | -0.033 |  |  |  |
|  |  |  |  |  |  |  | (-0.064, -0.002) |  |  |  |
|  | Fourth quintile |  |  |  |  |  | -0.039 |  |  |  |
|  |  |  |  |  |  |  | (-0.072, -0.006) |  |  |  |
|  | Highest quintile |  |  |  |  |  | -0.154 |  |  |  |
|  |  |  |  |  |  |  | (-0.189, -0.119) |  |  |  |
|  |  |  |  |  |  |  |  |  |  |  |
| **Tariff * Wealth index** | |  |  |  |  |  |  |  |  |  |
|  | Second quintile |  |  |  |  |  |  |  |  | -0.003 |
|  |  |  |  |  |  |  |  |  |  | (-0.007, 0.001) |
|  | Third quintile |  |  |  |  |  |  |  |  | -0.003 |
|  |  |  |  |  |  |  |  |  |  | (-0.007, 0.001) |
|  | Fourth quintile |  |  |  |  |  |  |  |  | 0.000 |
|  |  |  |  |  |  |  |  |  |  | (-0.004, 0.004) |
|  | Highest quintile |  |  |  |  |  |  |  |  | 0.011 |
|  |  |  |  |  |  |  |  |  |  | (0.007, 0.015) |
|  |  |  |  |  |  |  |  |  |  |  |
| ***Cluster-level predictors*** | |  |  |  |  |  |  |  |  |  |
|  | Urban residence | 0.431 | 0.49 | 0.379 | 0.431 | 0.441 | 0.432 | 0.431 | 0.44 | 0.43 |
|  |  | (0.392, 0.470) | (0.451, 0.529) | (0.340, 0.418) | (0.392, 0.470) | (0.402, 0.480) | (0.393, 0.471) | (0.392, 0.470) | (0.401, 0.479) | (0.391, 0.469) |
|  | Urban residence * GDP |  | -0.189 |  |  |  |  |  |  |  |
|  |  |  | (-0.205, -0.173) |  |  |  |  |  |  |  |
|  | Urban residence * FDI |  |  |  |  | -0.124 |  |  |  |  |
|  |  |  |  |  |  | (-0.151, -0.097) |  |  |  |  |
|  | Urban residence * Tariff |  |  |  |  |  |  |  | 0.012 |  |
|  |  |  |  |  |  |  |  |  | (0.008, 0.016) |  |
|  |  |  |  |  |  |  |  |  |  |  |
| ***National-level predictors*** | |  |  |  |  |  |  |  |  |  |
|  | GDP per capita | 0.002 | 0.158 | 0.081 | -0.001 | 0.014 | -0.002 | -0.006 | 0.02 | -0.008 |
|  |  | (-0.076, 0.080) | (0.080, 0.236) | (0.003, 0.159) | (-0.079, 0.077) | (-0.064, 0.092) | (-0.080, 0.076) | (-0.084, 0.072) | (-0.058, 0.098) | (-0.086, 0.070) |
|  | FDI (% GDP) |  |  |  | 0.014 | 0.028 | 0.065 |  |  |  |
|  |  |  |  |  | (-0.037, 0.065) | (-0.113, 0.169) | (0.010, 0.120) |  |  |  |
|  | Average annual tariff |  |  |  |  |  |  | 0.004 | 0 | 0.002 |
|  |  |  |  |  |  |  |  | (0.000, 0.008) | (-0.004, 0.004) | (-0.002, 0.006) |
| ***Random effects*** | |  |  |  |  |  |  |  |  |  |
|  | Level 1 (Individual) | 14.66 | 14.656 | 14.648 | 14.66 | 14.659 | 14.659 | 14.659 | 14.659 | 14.658 |
|  |  | (14.601, 14.719) | (14.597, 14.715) | (14.589, 14.707) | (14.601, 14.719) | (14.600, 14.718) | (14.600, 14.718) | (14.600, 14.718) | (14.600, 14.718) | (14.599, 14.717) |
|  | Level 2 (cluster) | 0.812 | 0.784 | 0.756 | 0.812 | 0.809 | 0.807 | 0.812 | 0.81 | 0.81 |
|  |  | (0.783, 0.841) | (0.757, 0.811) | (0.729, 0.783) | (0.783, 0.841) | (0.780, 0.838) | (0.780, 0.834) | (0.783, 0.841) | (0.781, 0.839) | (0.781, 0.839) |
|  | Level 3 (region) | 0.355 | 0.328 | 0.33 | 0.355 | 0.355 | 0.355 | 0.356 | 0.355 | 0.355 |
|  |  | (0.298, 0.412) | (0.275, 0.381) | (0.277, 0.383) | (0.298, 0.412) | (0.298, 0.412) | (0.298, 0.412) | (0.299, 0.413) | (0.298, 0.412) | (0.298, 0.412) |
|  | Level 4 (country) | 3.665 | 3.287 | 3.593 | 3.665 | 3.591 | 3.642 | 3.739 | 3.599 | 3.75 |
|  |  | (1.974, 5.356) | (1.770, 4.804) | (1.937, 5.249) | (1.975, 5.355) | (1.933, 5.249) | (1.962, 5.322) | (2.014, 5.464) | (1.935, 5.263) | (2.021, 5.479) |
|  |  |  |  |  |  |  |  |  |  |  |
| Constant | | 19.311 | 19.493 | 19.36 | 19.291 | 19.295 | 19.319 | 19.264 | 19.307 | 19.269 |
|  |  | (18.635, 19.987) | (18.848, 20.138) | (18.692, 20.028) | (18.611, 19.971) | (18.621, 19.969) | (18.641, 19.997) | (18.580, 19.948) | (18.635, 19.979) | (18.585, 19.953) |
|  |  |  |  |  |  |  |  |  |  |  |
| N |  | 507894 | 507894 | 507894 | 507894 | 507894 | 507894 | 507894 | 507894 | 507894 |
|  |  |  |  |  |  |  |  |  |  |  |
