# Supplementary material for: National Economic Development and Disparities in Body Mass Index: A Cross-Sectional Study of Data from 38 Countries
Source: PLoS One. 2014 Jun 11;9(6):e99327. doi: 10.1371/journal.pone.0099327 (PMC4053361; doi:10.1371/journal.pone.0099327)
Supplement: Table S6 — Odds ratios comparing underweight and normal weight and overweight and normal weight respondents by GDP and wealth and GDP and urban residence. (DOCX) [file pone.0099327.s006.docx]

**Table S6. Odds ratios comparing underweight and normal weight and overweight and normal weight respondents by GDP and wealth and GDP and urban residence**

|  |  | **Model 1. GDP** | **Model 2. GDP*urban** | **Model 3. GDP*Wealth** |
| --- | --- | --- | --- | --- |
|  |  | **OR** | **OR** | **OR** |
|  |  | **(95% CI)** | **(95% CI)** | **(95% CI)** |
| **Underweight** | |  |  |  |
| ***Individual-level predictors*** | |  |  |  |
| **Wealth index** | |  |  |  |
|  | Second quintile | 0.938 | 0.936 | 0.957 |
|  |  | (0.918, 0.958) | (0.916, 0.957) | (0.937, 0.978) |
|  | Third quintile | 0.882 | 0.879 | 0.904 |
|  |  | (0.864, 0.902) | (0.860, 0.898) | (0.883, 0.925) |
|  | Fourth quintile | 0.775 | 0.773 | 0.809 |
|  |  | (0.757, 0.793) | (0.757, 0.790) | (0.790, 0.828) |
|  | Highest quintile | 0.628 | 0.632 | 0.664 |
|  |  | (0.611, 0.646) | (0.615, 0.649) | (0.645, 0.684) |
| **GDP * Wealth index** | |  |  |  |
|  | Second quintile |  |  | 1.036 |
|  |  |  |  | (1.022, 1.050) |
|  | Third quintile |  |  | 1.040 |
|  |  |  |  | (1.024, 1.056) |
|  | Fourth quintile |  |  | 1.071 |
|  |  |  |  | (1.055, 1.088) |
|  | Highest quintile |  |  | 1.079 |
|  |  |  |  | (1.062, 1.096) |
|  |  |  |  |  |
| ***Cluster-level predictors*** | |  |  |  |
|  | Urban residence | 0.897 | 0.906 | 0.890 |
|  |  | (0.881, 0.913) | (0.890, 0.922) | (0.875, 0.906) |
|  | Urban residence * GDP | | 1.030 |  |
|  |  |  | (1.020, 1.041) |  |
|  |  |  |  |  |
| ***National-level predictors*** | |  |  |  |
|  | GDP per capita | 0.946 | 0.934 | 0.911 |
|  |  | (0.940, 0.951) | (0.927, 0.942) | (0.902, 0.920) |
|  |  |  |  |  |
| **Constant** |  | 0.050 | 0.049 | 0.050 |
|  |  | (0.039, 0.063) | (0.039, 0.062) | (0.040, 0.063) |
| **Overweight** | |  |  |  |
| ***Individual-level predictors*** | |  |  |  |
| **Wealth index** | |  |  |  |
|  | Second quintile | 1.188 | 1.278 | 1.241 |
|  |  | (1.160, 1.216) | (1.248, 1.308) | (1.210, 1.273) |
|  | Third quintile | 1.313 | 1.496 | 1.476 |
|  |  | (1.282, 1.344) | (1.462, 1.532) | (1.438, 1.514) |
|  | Fourth quintile | 1.605 | 1.781 | 1.872 |
|  |  | (1.567, 1.643) | (1.739, 1.823) | (1.825, 1.920) |
|  | Highest quintile | 2.413 | 2.455 | 2.718 |
|  |  | (2.353, 2.476) | (2.393, 2.518) | (2.645, 2.794) |
| **GDP * Wealth index** | |  |  |  |
|  | Second quintile |  |  | 0.968 |
|  |  |  |  | (0.958, 0.977) |
|  | Third quintile |  |  | 0.915 |
|  |  |  |  | (0.906, 0.924) |
|  | Fourth quintile |  |  | 0.862 |
|  |  |  |  | (0.853, 0.870) |
|  | Highest quintile |  |  | 0.746 |
|  |  |  |  | (0.739, 0.753) |
|  |  |  |  |  |
| ***Cluster-level predictors*** | |  |  |  |
|  | Urban residence | 1.505 | 1.637 | 1.474 |
|  |  | (1.482, 1.529) | (1.612, 1.663) | (1.451, 1.497) |
|  | Urban residence * GDP | | 0.821 |  |
|  |  |  | (0.816, 0.826) |  |
|  |  |  |  |  |
| ***National-level predictors*** | |  |  |  |
|  | GDP per capita | 1.397 | 1.543 | 1.589 |
|  |  | (1.391, 1.402) | (1.534, 1.553) | (1.576, 1.601) |
|  |  |  |  |  |
| **Constant** |  | 0.044 | 0.041 | 0.040 |
|  |  | (0.042, 0.047) | (0.039, 0.044) | (0.037, 0.042) |
|  |  |  |  |  |
| **N** |  | 697573 | 697573 | 697573 |

Model also adjusted for age (5-year groups), educational attainment (no/incomplete primary, complete primary/incomplete secondary, complete secondary and higher), marital status, and survey year (categorical).
