# Supplementary material for: National Economic Development and Disparities in Body Mass Index: A Cross-Sectional Study of Data from 38 Countries
Source: PLoS One. 2014 Jun 11;9(6):e99327. doi: 10.1371/journal.pone.0099327 (PMC4053361; doi:10.1371/journal.pone.0099327)
Supplement: Table S7 — Odds ratios comparing underweight and normal weight and overweight and normal weight respondents by FDI and wealth and FDI and urban residence. (DOCX) [file pone.0099327.s007.docx]

**Table S7. Odds ratios comparing underweight and normal weight and overweight and normal weight respondents by FDI and wealth and FDI and urban residence**

|  |  | **Model 1. FDI** | **Model 2. FDI*urban** | **Model 3. FDI*Wealth** |
| --- | --- | --- | --- | --- |
|  |  | **OR** | **OR** | **OR** |
|  |  | **(95% CI)** | **(95% CI)** | **(95% CI)** |
| **Underweight** | |  |  |  |
| ***Individual-level predictors*** | |  |  |  |
| **Wealth index** | |  |  |  |
|  | Second quintile | 0.927 | 0.923 | 0.925 |
|  |  | (0.907, 0.947) | (0.903, 0.943) | (0.905, 0.945) |
|  | Third quintile | 0.864 | 0.858 | 0.866 |
|  |  | (0.846, 0.883) | (0.840, 0.877) | (0.847, 0.885) |
|  | Fourth quintile | 0.752 | 0.748 | 0.777 |
|  |  | (0.735, 0.770) | (0.731, 0.766) | (0.759, 0.796) |
|  | Highest quintile | 0.603 | 0.602 | 0.655 |
|  |  | (0.587, 0.620) | (0.586, 0.619) | (0.637, 0.673) |
| **FDI * Wealth index** | |  |  |  |
|  | Second quintile |  |  | 1.011 |
|  |  |  |  | (0.988, 1.035) |
|  | Third quintile |  |  | 1.029 |
|  |  |  |  | (1.005, 1.054) |
|  | Fourth quintile |  |  | 1.113 |
|  |  |  |  | (1.087, 1.139) |
|  | Highest quintile |  |  | 1.270 |
|  |  |  |  | (1.240, 1.300) |
|  |  |  |  |  |
| ***Cluster-level predictors*** | |  |  |  |
|  | Urban residence | 0.916 | 0.939 | 0.908 |
|  |  | (0.900, 0.932) | (0.923, 0.956) | (0.893, 0.925) |
|  | Urban residence * GDP |  | 1.085 |  |
|  |  |  | (1.069, 1.103) |  |
|  |  |  |  |  |
| ***National-level predictors*** | |  |  |  |
|  | GDP per capita | 0.962 | 0.959 | 0.966 |
|  |  | (0.956, 0.967) | (0.953, 0.965) | (0.960, 0.971) |
|  | FDI (% GDP) | 0.816 | 1.005 | 0.793 |
|  |  | (0.808, 0.824) | (1.584, 0.638) | (0.784, 0.802) |
|  |  |  |  |  |
| **Constant** |  | 0.040 | 0.040 | 0.037 |
|  |  | (0.031, 0.050) | (0.031, 0.050) | (0.029, 0.047) |
| **Overweight** | |  |  |  |
| ***Individual-level predictors*** | |  |  |  |
| **Wealth index** | |  |  |  |
|  | Second quintile | 1.201 | 1.224 | 1.232 |
|  |  | (1.173, 1.229) | (1.195, 1.253) | (1.201, 1.264) |
|  | Third quintile | 1.339 | 1.383 | 1.425 |
|  |  | (1.308, 1.371) | (1.351, 1.416) | (1.392, 1.459) |
|  | Fourth quintile | 1.649 | 1.701 | 1.788 |
|  |  | (1.610, 1.688) | (1.661, 1.741) | (1.743, 1.834) |
|  | Highest quintile | 2.512 | 2.550 | 2.694 |
|  |  | (2.449, 2.577) | (2.486, 2.616) | (2.621, 2.769) |
| **FDI * Wealth index** | |  |  |  |
|  | Second quintile |  |  | 0.976 |
|  |  |  |  | (0.959, 0.994) |
|  | Third quintile |  |  | 0.915 |
|  |  |  |  | (0.899, 0.931) |
|  | Fourth quintile |  |  | 0.862 |
|  |  |  |  | (0.847, 0.878) |
|  | Highest quintile |  |  | 0.745 |
|  |  |  |  | (0.732, 0.758) |
|  |  |  |  |  |
| ***Cluster-level predictors*** | |  |  |  |
|  | Urban residence | 1.486 | 1.523 | 1.483 |
|  |  | (1.463, 1.509) | (1.500, 1.548) | (1.460, 1.506) |
|  | Urban residence * GDP |  | 0.852 |  |
|  |  |  | (0.844, 0.861) |  |
|  |  |  |  |  |
| ***National-level predictors*** | |  |  |  |
|  | GDP per capita | 1.385 | 1.387 | 1.383 |
|  |  | (1.380, 1.391) | (1.381, 1.392) | (1.377, 1.388) |
|  | FDI (% GDP) | 1.133 | 1.004 | 1.235 |
|  |  | (1.124, 1.142) | (0.664, 1.518) | (1.223, 1.247) |
|  |  |  |  |  |
| **Constant** |  | 0.051 | 0.049 | 0.054 |
|  |  | (0.048, 0.055) | (0.046, 0.052) | (0.051, 0.058) |
|  |  |  |  |  |
| **N** |  | 697573 | 697573 | 697573 |

Model also adjusted for age (5-year groups), educational attainment (no/incomplete primary, complete primary/incomplete secondary, complete secondary and higher), marital status, and survey year (categorical).
