# Supplementary material for: National Economic Development and Disparities in Body Mass Index: A Cross-Sectional Study of Data from 38 Countries
Source: PLoS One. 2014 Jun 11;9(6):e99327. doi: 10.1371/journal.pone.0099327 (PMC4053361; doi:10.1371/journal.pone.0099327)
Supplement: Table S8 — Odds ratios comparing underweight and normal weight and overweight and normal weight respondents by tariff rate and wealth and tariff rate and urban residence. (DOCX) [file pone.0099327.s008.docx]

**Table S8. Odds ratios comparing underweight and normal weight and overweight and normal weight respondents by tariff rate and wealth and tariff rate and urban residence**

|  |  | **Model 1.  Average tariff** | **Model 2.  Average tariff*urban** | **Model 3.  Average tariff*Wealth** |
| --- | --- | --- | --- | --- |
|  |  | **OR** | **OR** | **OR** |
|  |  | **(95% CI)** | **(95% CI)** | **(95% CI)** |
| **Underweight** | |  |  |  |
| ***Individual-level predictors*** | |  |  |  |
| **Wealth index** | |  |  |  |
|  | Second quintile | 0.929 | 0.929 | 0.930 |
|  |  | (0.909, 0.949) | (0.909, 0.949) | (0.910, 0.950) |
|  | Third quintile | 0.861 | 0.860 | 0.862 |
|  |  | (0.842, 0.879) | (0.842, 0.879) | (0.843, 0.880) |
|  | Fourth quintile | 0.745 | 0.745 | 0.752 |
|  |  | (0.728, 0.763) | (0.727, 0.762) | (0.735, 0.770) |
|  | Highest quintile | 0.590 | 0.590 | 0.607 |
|  |  | (0.574, 0.606) | (0.574, 0.607) | (0.590, 0.623) |
| **Average tariff * Wealth index** | |  |  |  |
|  | Second quintile |  |  | 0.999 |
|  |  |  |  | (0.997, 1.001) |
|  | Third quintile |  |  | 0.999 |
|  |  |  |  | (0.997, 1.001) |
|  | Fourth quintile |  |  | 0.996 |
|  |  |  |  | (0.994, 0.998) |
|  | Highest quintile |  |  | 0.987 |
|  |  |  |  | (0.985, 0.989) |
|  |  |  |  |  |
| ***Cluster-level predictors*** | |  |  |  |
|  | Urban residence | 0.931 | 0.935 | 0.928 |
|  |  | (0.914, 0.947) | (0.919, 0.952) | (0.912, 0.944) |
|  | Urban residence * Average tariff |  | 0.997 |  |
|  |  |  | (0.995, 0.999) |  |
|  |  |  |  |  |
| ***National-level predictors*** | |  |  |  |
|  | GDP per capita | 0.953 | 0.952 | 0.953 |
|  |  | (0.948, 0.959) | (0.947, 0.958) | (0.948, 0.959) |
|  | Average tariff (%) | 1.036 | 1.000 | 1.037 |
|  |  | (1.036, 1.036) | (0.932, 1.073) | (1.035, 1.039) |
|  |  |  |  |  |
| **Constant** |  | 0.048 | 0.048 | 0.048 |
|  |  | (0.038, 0.061) | (0.038, 0.061) | (0.038, 0.060) |
| **Overweight** | |  |  |  |
| ***Individual-level predictors*** | |  |  |  |
| **Wealth index** | |  |  |  |
|  | Second quintile | 1.189 | 1.196 | 1.182 |
|  |  | (1.161, 1.217) | (1.168, 1.224) | (1.154, 1.210) |
|  | Third quintile | 1.317 | 1.338 | 1.323 |
|  |  | (1.286, 1.348) | (1.307, 1.370) | (1.292, 1.355) |
|  | Fourth quintile | 1.614 | 1.639 | 1.636 |
|  |  | (1.577, 1.653) | (1.601, 1.678) | (1.598, 1.675) |
|  | Highest quintile | 2.435 | 2.450 | 2.460 |
|  |  | (2.374, 2.498) | (2.388, 2.513) | (2.398, 2.523) |
| **Average tariff * Wealth index** | |  |  |  |
|  | Second quintile |  |  | 0.997 |
|  |  |  |  | (0.995, 0.999) |
|  | Third quintile |  |  | 1.000 |
|  |  |  |  | (0.998, 1.002) |
|  | Fourth quintile |  |  | 1.004 |
|  |  |  |  | (1.002, 1.006) |
|  | Highest quintile |  |  | 1.014 |
|  |  |  |  | (0.998, 1.016) |
|  |  |  |  |  |
| ***Cluster-level predictors*** | |  |  |  |
|  | Urban residence | 1.499 | 1.516 | 1.499 |
|  |  | (1.476, 1.523) | (1.492, 1.540) | (1.476, 1.523) |
|  | Urban residence * GDP |  | 1.014 |  |
|  |  |  | (1.012, 1.016) |  |
|  |  |  |  |  |
| ***National-level predictors*** | |  |  |  |
|  | GDP per capita | 1.394 | 1.397 | 1.392 |
|  |  | (1.388, 1.399) | (1.391, 1.402) | (1.387, 1.398) |
|  | Average tariff (%) | 0.996 | 1.000 | 0.989 |
|  |  | (0.996, 0.996) | (1.022, 0.979) | (0.987, 0.991) |
|  |  |  |  |  |
| **Constant** |  | 0.045 | 0.044 | 0.046 |
|  |  | (0.042, 0.048) | (0.041, 0.047) | (0.043, 0.049) |
|  |  |  |  |  |
| **N** |  | 697573 | 697573 | 697573 |

Model also adjusted for age (5-year groups), educational attainment (no/incomplete primary, complete primary/incomplete secondary, complete secondary and higher), marital status, and survey year (categorical).
